# Temperature‑Dependent Friction, Wear, and Life of MoS₂ Dry Film Lubricants for Space Mechanisms: A Comprehensive Review

**DOI:** 10.1007/s11249-025-02052-6

**Published:** 2025-08-01

**Authors:** Abrar Faiyad, Daniel Miliate, Samuel Leventini, Jeffrey R. Lince, Ashlie Martini

**Affiliations:** 1 Department of Mechanical and Aerospace Engineering, University of California Merced, Merced, USA; 2 Space Tribology Consulting, Inc., Culver City, USA

**Keywords:** Dry film lubricants, Molybdenum disulfide, Temperature dependence, Space tribology

## Abstract

**Abstract:**

Molybdenum Disulfide (MoS_2_) is the most widely used dry film lubricant (DFL) for moving mechanical assemblies that operate in space. For these applications, the MoS_2_ must provide low friction and wear across a range of temperatures. The temperature dependence of MoS_2_ tribological behavior has been studied previously. However, the number of temperatures and conditions that can be tested in a single study is necessarily limited, making it difficult to predict or understand the performance of DFLs more broadly. To address this, this review article summarizes and analyzes the results from prior studies of temperature-dependent tribological behavior of MoS_2_-based dry film lubricants. Friction, wear, and wear life data are compiled into a plot matrix and trends are identified. Then, the mechanisms that have been proposed to explain observed trends are summarized. Finally, gaps in the knowledge and opportunities for future work are discussed such that researchers can build on existing studies to enhance the reliability and performance of MoS_2_-based dry film lubricants in space environments.

**Graphical Abstract:**

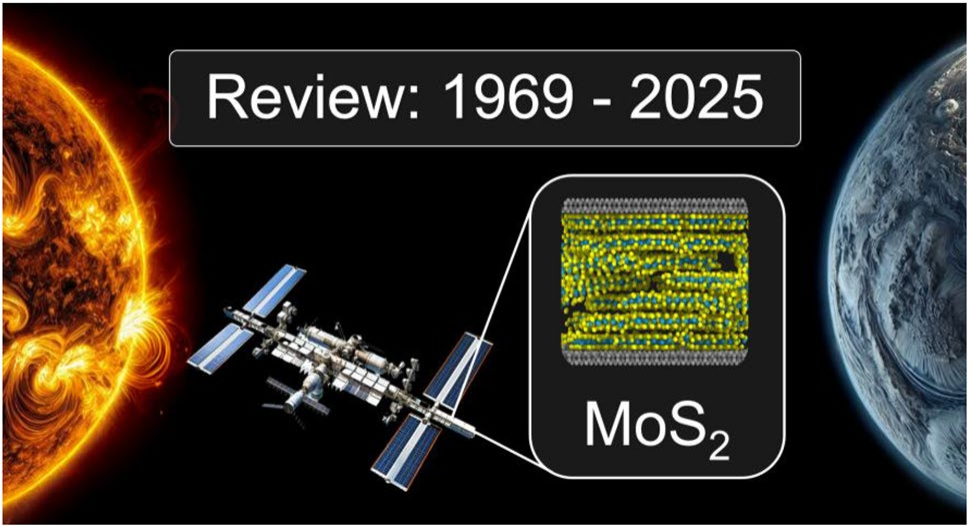

**Supplementary Information:**

The online version contains supplementary material available at 10.1007/s11249-025-02052-6.

## Introduction

This article summarizes and analyzes previous research on the temperature-dependence of dry film lubricants based on Molybdenum Disulfide (MoS_2_) [1–21]. The timing of the review is motivated by the exponential increase in the number of objects launched into space in recent years [22]. With growing interest in space exploration, new engineering challenges must be addressed. One such challenge is the design or selection of lubricants for moving mechanical assemblies (MMAs) on space missions. Lubricants reduce friction and wear, which directly impacts the energy consumption and the operation life of the MMA. However, while most lubricants here on Earth are liquids, they are not always viable for MMAs in space [23].

One significant challenge for liquid lubricants is the wide range of temperatures at which they may operate during a space mission. For example, the surface of the moon can reach 90 K at night and 400 K during the day. On other celestial bodies, temperatures can be even more extreme; for example, 70 K on the moon Europa and 525 K on Mercury [24]. Very cold environments are challenging for MMAs since heaters must be employed to prevent the viscosity of liquid lubricants from increasing excessively; such heaters are heavy and consume energy, which reduces overall science payload and decreases operational time. Conversely, in hot conditions the viscosity of liquid lubricants decreases rapidly and evaporative loss increases, both of which may lead to accelerated wear of the contacting surfaces. Even at moderate temperatures, liquid lubricants can evaporate due to vacuum pressure, risking contamination of sensors [24]. To overcome the limitations of liquid lubricants, dry film lubricant (DFL) coatings are a viable alternative [23]. DFLs are solid coatings that overcome many of the challenges inherent to liquid lubricants.

DFL coatings have been extensively studied [23], and MoS_2_ stands out as the most widely used DFL for space MMAs because it excels in extreme environments including vacuum and at a range of temperatures and Hertzian contact stresses. MoS_2_ is a lamellar material with strong intralayer bonding but weak interlayer bonding that allows for easy shear and low friction and wear [24]. To further improve the properties of MoS_2_ DFLs, they can be doped with additional elements [23, 24]. Undoped and doped MoS_2_ DFLs (together referred to as MoS_2_-based DFLs) have been used successfully for MMAs on numerous space missions and satellites [25, 26].

Despite the proven success of MoS_2_-based DFLs, significant challenges remain in fully characterizing their tribological performance. This characterization typically involves measuring properties such as friction, wear, and wear life using tribometers on Earth. However, it is essential that tribometer tests (or tribotests) accurately replicate the environmental and operational conditions encountered during an MMA’s entire journey to space, from pre-flight testing and transport, to launch, and then operation in space. Particularly, tribotests must simulate the wide temperature range an MMA may experience during a space mission.

Many previous studies have used tribotests to characterize the tribological properties of MoS_2_-based DFLs [23], some of which focused specifically on temperature dependence [1–21]. However, most of these studies included only a limited range of temperatures and environments or evaluated just one or a few different MoS_2_-based coatings. The limited scope possible in individual studies makes it difficult to predict the performance of DFLs intended for the wide range of temperatures faced by MMAs for space applications. To address these issues, this review summarizes the temperature dependence of MoS_2_-based DFLs to identify and quantify trends. The mechanisms behind these trends are discussed and, finally, gaps and opportunities for future research are suggested.

## Summary of Methods and Data Selection Process

This review comprises data and results from previous studies on MoS_2_-based DFLs tribotested at two or more temperatures. Data was obtained either directly from reported values or estimated from plots using WebPlotDigitizer [27]. To understand the data that is compiled and analyzed later in the review, we start with a summary of the sample preparation and tribotesting methods used in previous studies. Detailed information about the test parameters used is available in a spreadsheet included as part of the Supporting Information (SI). Distributions of the test operating and environmental conditions are also shown graphically as stacked histograms in Figs. S1–S9.

MoS_2_-based DFLs are applied to a substrate as a coating. The studies reviewed here used either burnishing [1, 16] or sputtering [2–15, 17–21] for this purpose. Sputtering is most common in these studies and is often preferred for space coatings because it provides better adhesion and also produces nanostructures that lower friction and optimize material properties [17]. Sputtering also gives better control of coating thickness, which has been shown to affect the performance of MoS_2_-based DFLs [17, 28]. The coating thickness in the studies reviewed here ranged from 0.2 to 2 µm, with the median coating thickness being 1 µm. MoS_2_ can be deposited with other elements to produce doped DFLs. Doped MoS_2_ DFLs have been shown to exhibit better tribological performance than their undoped counterparts in multiple previous studies, as discussed later. The studies reviewed here used various dopants at a range of concentrations in the DFL. Although some studies did not report the dopant weight percentage, among those that did report it, weight percentages ranged from 2 wt.% to 50 wt/%, with a median value of 11 wt.%. When available, the dopant weight percentage and coating manufacturer are given in the spreadsheet in the SI.

In some cases, a thin interlayer, also known as an adhesion layer, is applied to the substrate prior to MoS_2_ deposition to enhance bonding. The bonding strength between the coating and the surface depends on the substrate material [29, 30] as well as the roughness of the substrate surface [31]. The average roughness of substrates in the studies reviewed here ranged from 21 nm to 1 μm, with a median value of 30 nm. Substrates used in the studies reviewed here were usually bearing steel (such as 440C or 52100), although a few studies used other metal alloys or silicon nitride. The counterbody typically comprised the same or similar material as the substrate.

Tribotests involve two bodies pressed together and in relative motion. Studies selected for this review performed tribotests with single point contact in sliding. The point contact is typically formed by a ball or pin pressed against DFL-coated plate or disk; unless explicitly mentioned, studies reviewed here had those geometries. The two bodies are pressed together at a prescribed normal load and the nominal Hertzian contact pressure is calculated based on the load, geometry, and substrate material. The sliding could be linear reciprocating where one of the two contacting bodies moves back and forth along a linear path, or rotary unidirectional tests where one of the two bodies, usually the DFL-coated substrate, rotates. Linear speeds reported in the studies reviewed here ranged from 3 to 6000 mm/s, with a median speed of 733 mm/s, and the contact pressures ranged from 176 kPa to 1.86 GPa, with a median of 350 MPa. The exception to the conditions described above is one study [5] performed using atomic force microscopy (AFM) in which linear reciprocating sliding occurred between a nanoscale probe and the DFL. In that case, no external load was applied, although adhesion resulted in a non-zero normal force such that the resulting contact pressures are difficult to quantify, and speeds were on the order of a nanometer per second.

Tribometers used for testing DFLs often include enclosures in which the temperature and environment can be controlled. Usually, temperature is controlled by either adding or removing heat through direct contact with the bottom of the substrate or through the walls of the chamber. Reference [10] achieved extremely low temperatures by submerging the sample in either liquid nitrogen (LN_2_), liquid helium (LHe), or liquid hydrogen (LH_2_). Temperatures reported in the studies reviewed here ranged from 4.2 to 872 K. Enclosures are also used to control the chemical composition of the environment, specifically the amount of oxygen and/or the relative humidity (RH%). In the reviewed studies, tests run in open air had relative humidities between 20 and 45 RH%; in controlled humidity tests, humidity as high as 70 RH% was achieved. These conditions reflect the environments DFLs may encounter during pre-flight preparation, transport, or storage. To mimic the low oxygen and humidity conditions of space, testing can be performed either in vacuum or in an inert gas environment. Vacuum testing most accurately captures space conditions but is expensive and time consuming. The alternative is to flood the enclosure with inert gas, the most common inert gas being gaseous nitrogen (GN_2_).

The key performance metrics reported in tribological tests are the coefficient of friction (CoF), specific wear rate (SWR), and wear life. The CoF is the ratio of measured lateral force to applied normal force and is an indicator of resistance to sliding and wasted energy. Material removal can be quantified by wear volume, wear rate (volume per distance), or SWR (volume per distance per applied load). Lastly, wear life, also called endurance in some literature, is typically identified from an irreversible increase in friction that is assumed to indicate failure of the coating. Wear life can be reported in terms of cycles, time, or sliding distance. While both SWR and wear life are measures of material removal, they are distinct because they depend on different factors. Particularly, while both parameters depend on operating conditions and environment, wear life also depends on coating thickness and coating-substrate adhesion. Further, the rate of material removal from a DFL is not constant over the life of the coating, so SWR cannot be used to calculate wear life, or vice versa. In this review, we report CoF unitless, SWR in units of mm^3^/(N m), and wear life in units of cycles. If material removal was reported as wear volume or wear rate, the SWR was calculated based on reported test conditions. If wear life was reported as time or sliding distance, those values were converted to cycles. Some long duration tests were terminated before failure. The reported cycles are recorded in the SI but not included as a wear life result.

## Summary and Categorization of All Results

**Fig. 1:**
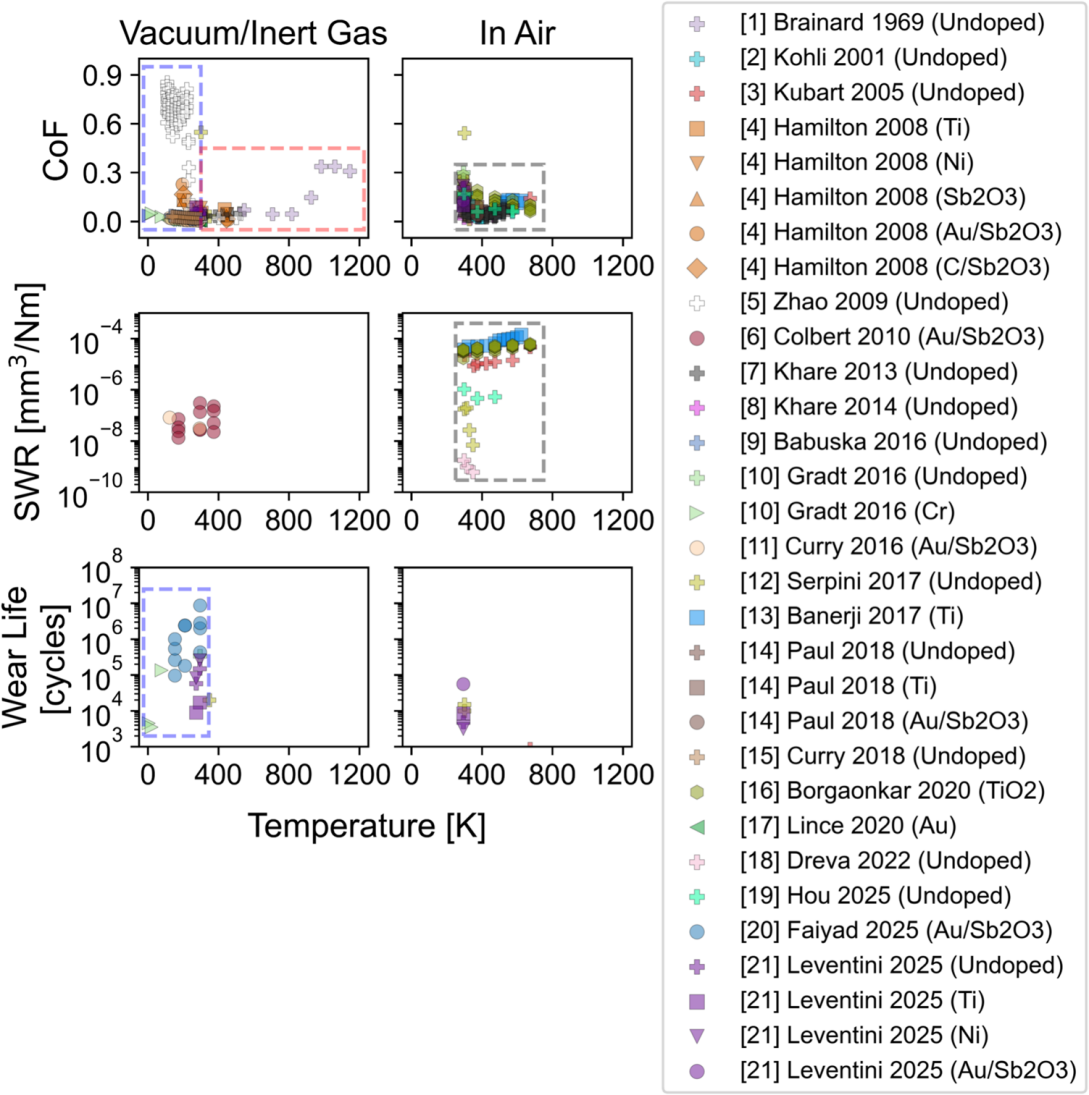
Data reported in previous studies are compiled into six plots arranged in a 2 × 3 grid, comparing friction and wear performance under vacuum/inert (left column) and in air (right column) environments. The rows display CoF, SWR, and wear life. Data points are distinguished by color, representing literature references, and shape, indicating dopants used, if any. The plots have been subdivided into three types of tests—CoF and wear life below 300 K in vacuum/inert gas (blue boxes), CoF above 270 K in vacuum/inert gas (red box), and CoF and SWR above 270 K in humid/dry air (grey boxes). The SWR and wear life data is replotted in SI (Figs. S10–S15) on a linear scale for reference (Color figure online)

Figure 1 presents six plots, comparing friction and wear performance as a function of temperature in different environmental conditions. The columns represent two types of environments: the first column includes data from experiments conducted in either an inert gas environment or a vacuum chamber, while the second column shows results from tests performed in air across a wide range of relative humidities. The three rows correspond to the three key tribological performance metrics: the CoF in the first row, SWR in the second row, and wear life in the third row.

Data points are represented using both color and shape where color indicates the references from which the data were sourced while shapes denote the dopants used, if any. For tests reported as being performed at “room temperature”, results are plotted in Fig. 1 at 294.5 K. At any given temperature, there is a scatter in the data, due to factors in coating composition, substrate material, environmental conditions, and experimental parameters. However, consistent trends can still be identified.

To analyze the trends observed in Fig. 1, we divide the data into three regions. The first set of data that will be discussed is CoF and wear life measured below 300 K in vacuum or inert gas, identified by the purple dashed line boxes in Fig. 1. Limited SWR data is available in these conditions. The next set of data is friction measured at temperatures above 270 K in vacuum or inert gas, identified by the pink dashed line box in the figure. There is no SWR or wear life data reported in these conditions. Lastly, friction and SWR data collected above 270 K in air, identified by the grey dashed line box in Fig. 1, will be discussed. Data measured in air is only available for temperatures above 270 K because, below this temperature, condensed water (ice) would likely dominate the tribological performance of the coating.

In the following sections, the data in these three regions will be analyzed. The data in the dashed boxes in Fig. 1 will be re-plotted in the subsequent sections such that individual references can be differentiated. Then, trends will be identified and described. Lastly, the mechanisms that have been proposed to underly these trends will be discussed.

## Friction and Wear Life Below 300 K in Vacuum/Inert Gas

### Identification of Trends Below 300 K in Vacuum/Inert Gas

**Fig. 2:**
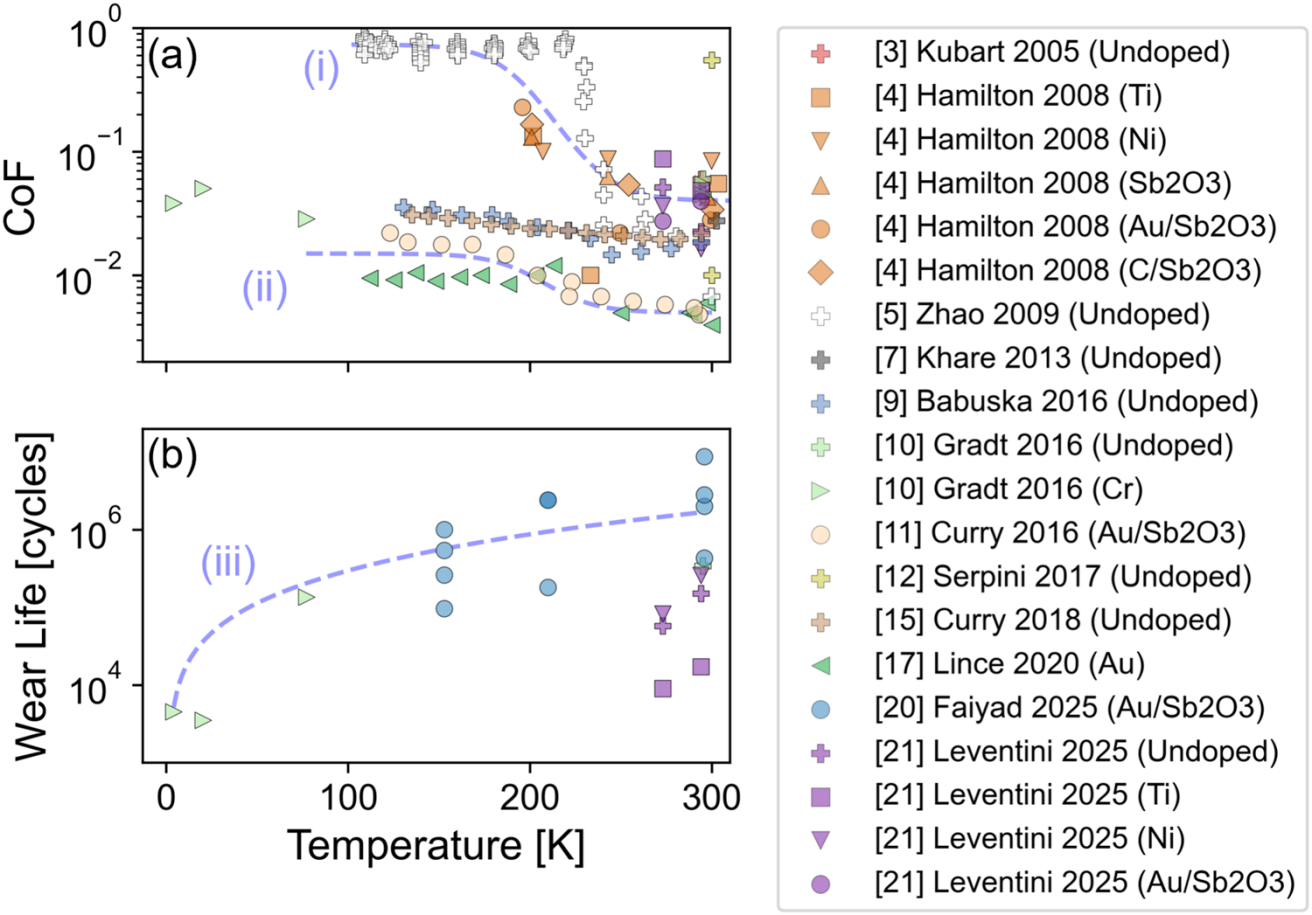
A focused view of results from vacuum or inert gas conditions below 300 K, showing **a** CoF and **b** wear life. The y-axes are on a logarithmic scale to highlight subtle temperature-dependent variations in CoF and wear life. Trendlines (i)– (iii) are included as a visual guide only and were not fit to data. Generally, performance deteriorates, i.e., CoF increases and wear life decreases, with decreasing temperature

Figure 2a shows the CoF of MoS_2_-based DFLs reported in the literature from tribotests performed at temperatures below 300 K in vacuum or inert gas environments. Figure 2b shows the temperature-dependent wear life in these conditions. Generally, friction coefficient increases and wear life decreases as temperature decreases below room temperature. This indicates cold temperatures adversely affect the tribological performance of MoS_2_-based DFLs.

The CoF data shown in Fig. 2a can be separated into two groups, differentiated by two dashed trend lines generated for visual aid only (i.e., not fit to data). The first trendline is labeled (i). This CoF data starts at greater than 0.02 at room temperature, then increases rapidly when the temperature reaches around 220 K, and finally exceeds 0.1 at temperatures below 200 K. This trend is mainly supported by data from [4, 5], although their methodologies differ. Reference [4] reports linear reciprocating pin-on-disk experiments on five different MoS_2_-based DFLs while Reference [5] is an AFM-based study on undoped MoS_2_. Reference [5] used an ultra-high vacuum chamber at 10^−10^ Torr to achieve low humidity and Reference [4] achieved an inert environment using GN_2_.

The second trendline in Fig. 2a is labeled (ii). This highlights another group of references where the CoF starts below 0.02 at room temperature but increases at lower temperatures, particularly temperatures below 220 K. For this data, even at the coldest temperature reported, 4.2 K, the friction coefficient is still less than 0.1. This trend is demonstrated by [9–11, 15, 17]. Reference [10] performed tests in liquid He (4.2 K), H_2_ (20 K), and N_2_ (77 K). References [9, 11] used a tribometer placed inside a GN_2_-purged chamber with < 10 ppm O_2_ and < 0.5 ppm H_2_O. References [15, 17] were performed in vacuum of ~ 1 × 10^−8^ Torr. Reference [15] reported an increase in CoF with decreasing temperature but observed a more gradual change as opposed to the sharp increase at 220 K.

Both trendlines in Fig. 2a exhibit similar S-shape profiles where the CoF increases sharply at a transition temperature around 220 K, although the CoF magnitudes differ. To highlight the similarity of the trends and transition temperature, min–max scaling was applied to the CoF data such that the minimum was set to 0 and maximum was set to 1 for each paper. The min–max scaled CoF, plotted in Fig. S16, shows that data from [4, 5, 9, 11, 17] are all well-described by a single trend line in the scaled plot that increases rapidly around 220 K.

The wear life data in Fig. 2b comprises data from [10, 20, 21]. References [20, 21] both ran tribotests under GN_2_ environment while Reference [10] tested in submerged liquid He, H_2_, or N_2_. The scatter in the data reflects inconsistencies in the measured wear life as well as the effect of different testing parameters. Particularly, Reference [20] reported that wear life tests were performed at < 0.5 RH% while Reference [21] reported that the humidity was < 5 RH% during tests. Despite this scatter and the relatively small amount of data available, these results show that wear life decreased by orders of magnitude as the temperature decreased from room temperature to 4.2 K.

Some studies reported the results of long running tribotests that were terminated before the failure criterion for wear life was met (data not shown in Fig. 2b). Tests may be terminated when a predetermined maximum number of cycles [20] or runtime [21] is reached. In such cases, the reported number of cycles represents a minimum wear life measurement, which may still be useful as a performance metric. Reference [20] reported five tests run at room temperature that were stopped at 5 × 10^6^ cycles while all tests run at 200 and 250 K reached the failure criterion for wear life before 5 × 10^6^ cycles. Reference [21] reported that tests with Au/Sb_2_O_3_-doped MoS_2_ DFLs were stopped at 8 × 10^5^ cycles at both room temperature and 273 K while tests with undoped, Ti-doped, and Ni-doped coatings reached the failure criterion for wear life before 8 × 10^5^ cycles at either temperature.

While most studies below room temperature in vacuum or inert gas reported CoF or wear life, two studies measured SWR in these conditions (data shown in Fig. 1 but not replotted in Fig. 2). Reference [11] reported that the SWR of Au/Sb_2_O_3_ doped MoS_2_ increased with decreasing temperature while Reference [6] reported an opposing trend in which SWR decreased at colder temperatures.

### Mechanisms Proposed in Literature Below 300 K in Vacuum/Inert Gas

At cold temperatures in vacuum or inert gas environments, MoS_2_-based DFLs exhibit a deterioration in tribological performance. Several mechanisms have been proposed to explain this trend.

**Fig. 3:**
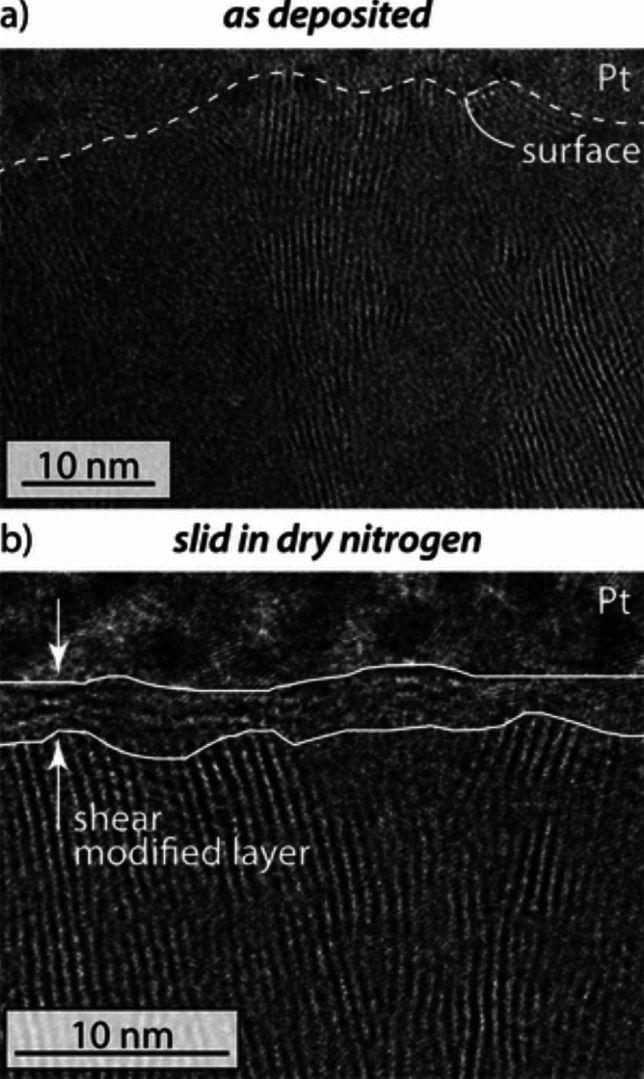
TEM bright-field images of a MoS_2_ DFL **a** as-deposited and **b** after sliding in dry GN_2_ showing a shear-modified layer of basally oriented material. Reproduced with permission [35]

Studies have proposed that cold temperatures can adversely affect the friction and wear of MoS_2_ DFLs by disrupting the formation of stable MoS_2_ lamella. Most MoS_2_-based DFLs are initially amorphous after deposition, but then form ordered, basally oriented lamella during sliding [32–35]; this shear-induced material evolution is shown in Fig. 3. The nucleation and crystallization of amorphous MoS_2_ was shown to depend on temperature, load, and sliding in molecular dynamics (MD) simulations [36]. When ordered MoS_2_ layers are transferred from the DFL to an uncoated counterbody, they create what is referred to as a transfer film [11, 37–40]. The formation of the basally oriented lamella and the transfer film are key steps to enabling the easy shear ability of MoS_2_ [41]. Reference [11] suggesting that, at low temperatures, the ability to form and maintain basally oriented layers was hindered, leading to higher friction. Reference [11] also hypothesized that an increase in wear was a result of the transfer film being less stable at lower temperatures, supported by scanning electron microscopy images of debris or flake accumulation at the wear track edges. A simulation-based analysis in [20] suggested that basally oriented layer formation can be impeded by the presence of small, less lubricious MoS_2_ flakes that form due to the brittle facture of MoS_2_ at cold temperatures. Reference [15] performed simulations to calculate the energy barrier for sliding of different sizes of MoS_2_ flakes and reported that smaller flakes have a higher energy barrier than larger flakes. Lastly, Reference [10] proposed that a thermal expansion mismatch between the DFL and the substrate can cause thermal stress in the DFL which may lead to early failure at cold temperatures.

**Fig. 4:**
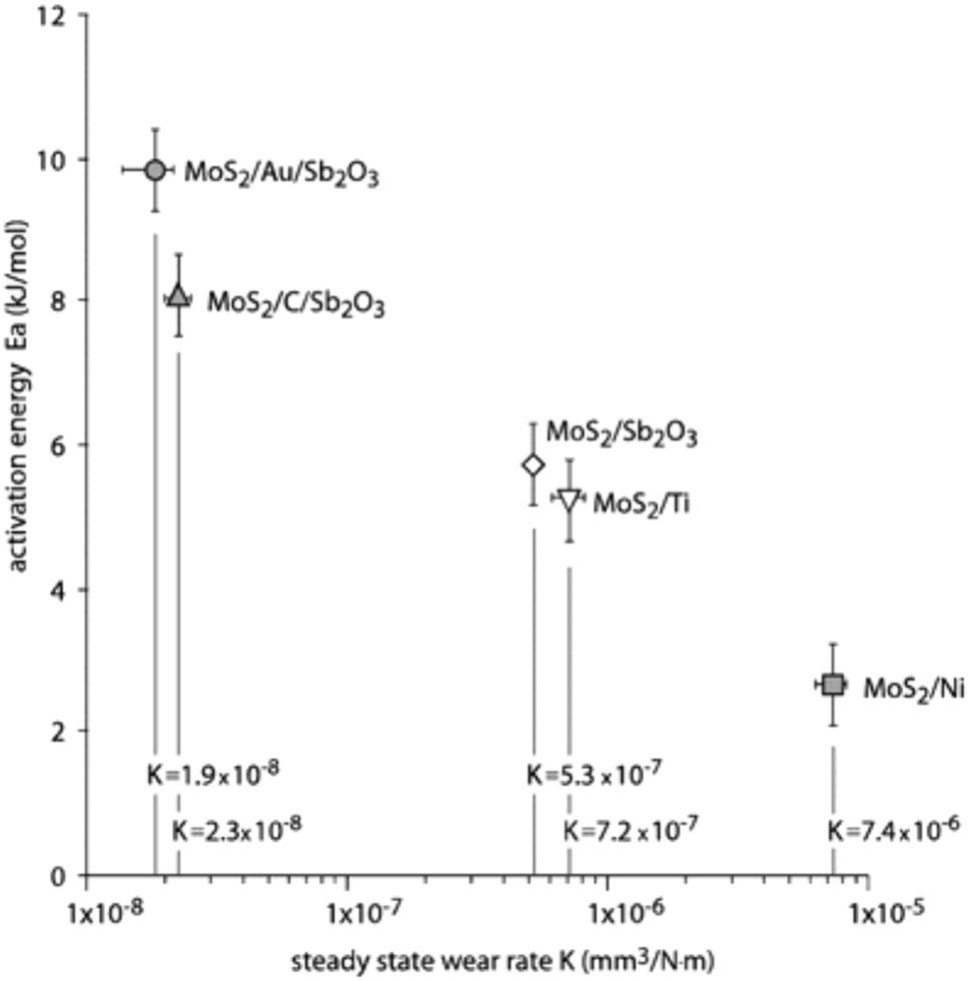
Activation energy calculated from temperature-dependent friction data as a function of SWR for five different MoS_2_-based DFLs. The higher wear rate DFLs has lower activation energy corresponding to a weaker dependence on temperature. Reproduced with permission [4]

Some proposed mechanisms occur through thermally activated processes. For example, relative motion between an AFM tip and MoS_2_ could be described by stress-assisted, activated propagation of dislocations, where the tip-surface contact is viewed as a dislocation on the substrate surface [5]. Another possible mechanism involves a dissipative component of the sliding interaction that could exhibit a thermally activated form, analogous to shear flow in liquids and glasses [4, 5]. These concepts have been used to explain the increase in friction with decreasing temperature, especially the sharp increase in friction observed around 220 K in several studies. Reference [5] referred to this temperature as a transition between thermal (above 220 K) and athermal (below 220 K) friction behavior. In [4], the CoF data from linear reciprocating tribotests was fit to an Arrhenius equation for thermally activated processes and used to calculate an activation energy. Activation energy was also calculated in [9, 11], although the magnitudes differed among [4, 9, 11]. Reference [4] reported that activation energy, and therefore temperature dependence, was lower for DFLs with higher SWR, as shown in Fig. 4. Based on this observation, it was suggested that friction will only be dependent on temperature in very low wear conditions in which the surface potential is the dominant energetic barrier to sliding [4].

**Fig. 5:**
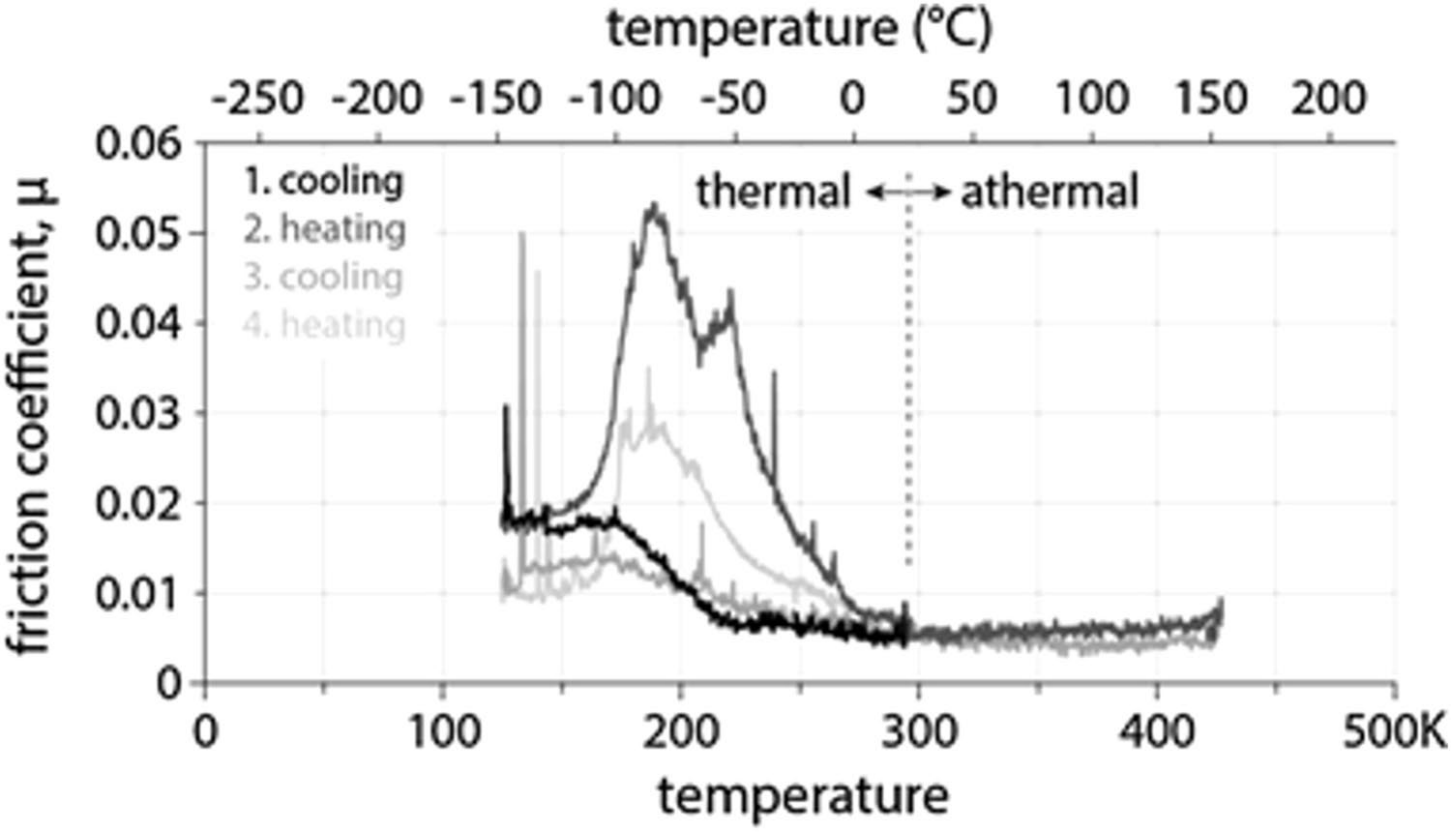
CoF from temperature cycling tests exhibits hysteresis. The CoF increases dramatically around 220 K in the heating cycles compared to the relatively small increase observed in the cooling cycles. The hysteresis is reduced with increasing number of cycles. Reproduced with permission [11]

Reference [11] performed temperature cycling tests and observed hysteresis where the friction during cooling was lower than the friction during heating, as seen in Fig. 5. The hysteresis decreased with increasing number of thermal cycles. Fitting this data to an Arrhenius function resulted in a cycle-dependent activation energy. Reference [11] hypothesized that the hysteresis between heating and cooling cycles was due to the expression of doping species (Au and Sb_2_O_3_), although water adsorption was also identified as a possible explanation.

## Friction Above 270 K in Vacuum/Inert Gas

### Identification of Trends Above 270 K in Vacuum/Inert Gas

**Fig. 6:**
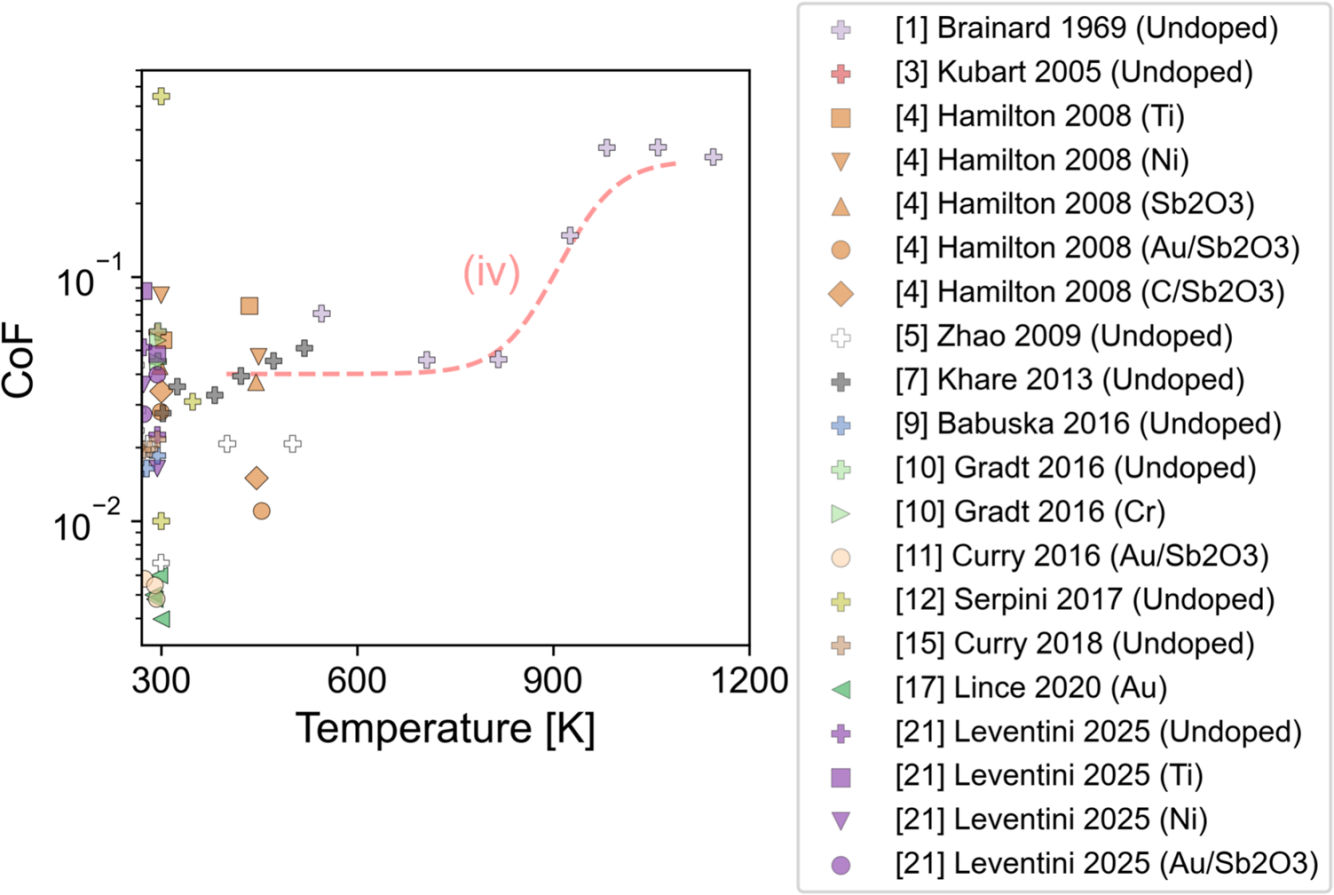
A focused view of the CoF measured in vacuum/inert gas conditions between 270 and 1200 K. The y-axis is presented on a logarithmic scale. The trendline (iv) is included as a visual guide only. There is no consistent trend at moderate temperatures but one study showed the CoF increased sharply around 800 K

Figure 6 shows the CoF of MoS_2_-based DFLs reported in literature from tribotests performed at temperatures above 270 K in vacuum or inert gas. Most of the data is around room temperature since that is often used as the reference temperature. Despite differences in methodologies between studies, the CoF is consistently below 0.1 at room temperature, indicating that the DFLs exhibit good performance irrespective of other factors. One test by [12] reported a high CoF of 0.54 at room temperature in GN_2_. This test corresponds to a coating that was intentionally oxidized prior to testing, so the high CoF represents the worst-case scenario for friction in a GN_2_ environment.

Between 270 and 540 K, some studies report a slight decrease in CoF with increasing temperature. This trend is supported by [4, 21]. Reference [4] tested several different doped MoS_2_ coatings in GN_2_ and found that the CoF of Ni-doped, Sb_2_O_3_-doped, Au/Sb_2_O_3_-doped, and C/Sb_2_O_3_-doped MoS_2_ decreased with increasing temperature up to 450 K. Reference [21] performed tests under GN_2_ and observed a decrease in CoF for Ni-doped, Ti-doped, and undoped MoS_2_ when increasing temperature from 270 K to room temperature.

However, other data sets exhibited a slight increase in CoF between 270 and 540 K. In Reference [4], the CoF of the Ti-doped MoS_2_ coating increased slightly with increasing temperature, although the CoF remained below 0.1 up to 450 K. Reference [21] reported an increase in CoF for Au/Sb_2_O_3_-doped MoS_2_ between 270 K and room temperature. Reference [7] reported the results of tests in GN_2_ and showed that the CoF increased for undoped MoS_2_ from 0.027 at 300 K to 0.05 at 520 K. Lastly, Reference [12] performed tests under GN_2_ and reported that the CoF was similar at room temperature and 350 K for undoped MoS_2_ during the first 10^4^ cycles. After 10^4^ cycles, the CoF at 350 K started to increase whereas the CoF at room temperature remained unchanged for 2.5 × 10^4^ cycles at which point the test was stopped.

For temperatures above 540 K, the only available CoF dataset was reported by [1]. The trend line (iv) in Fig. 6 between 540 and 1150 K is based on data collected from ball-on-disk tests performed on pure MoS_2_ powder samples that were burnished onto a cobalt alloy surface. Reference [1] reported that the coatings provided low CoF (< 0.1) at temperatures from 540 to 800 K. From 800 to 980 K, the study reported a sudden increase in CoF. Above 980 K, the CoF was very high, and the coating was reported to be no longer effective as a lubricant.

### Mechanisms Proposed in Literature Above 270 K in Vacuum/Inert Gas

At temperatures above 270 K in vacuum or inert gas environments, References [4, 5, 7, 12, 21] reported low CoF that changed little with temperature up to 540 K. They proposed that the MoS_2_-based DFLs had sufficient thermal energy to facilitate formation and transfer of basally oriented MoS_2_ lamella, similar to the mechanisms proposed for temperatures above 220 K in Sect. 4.2.

**Fig. 7:**
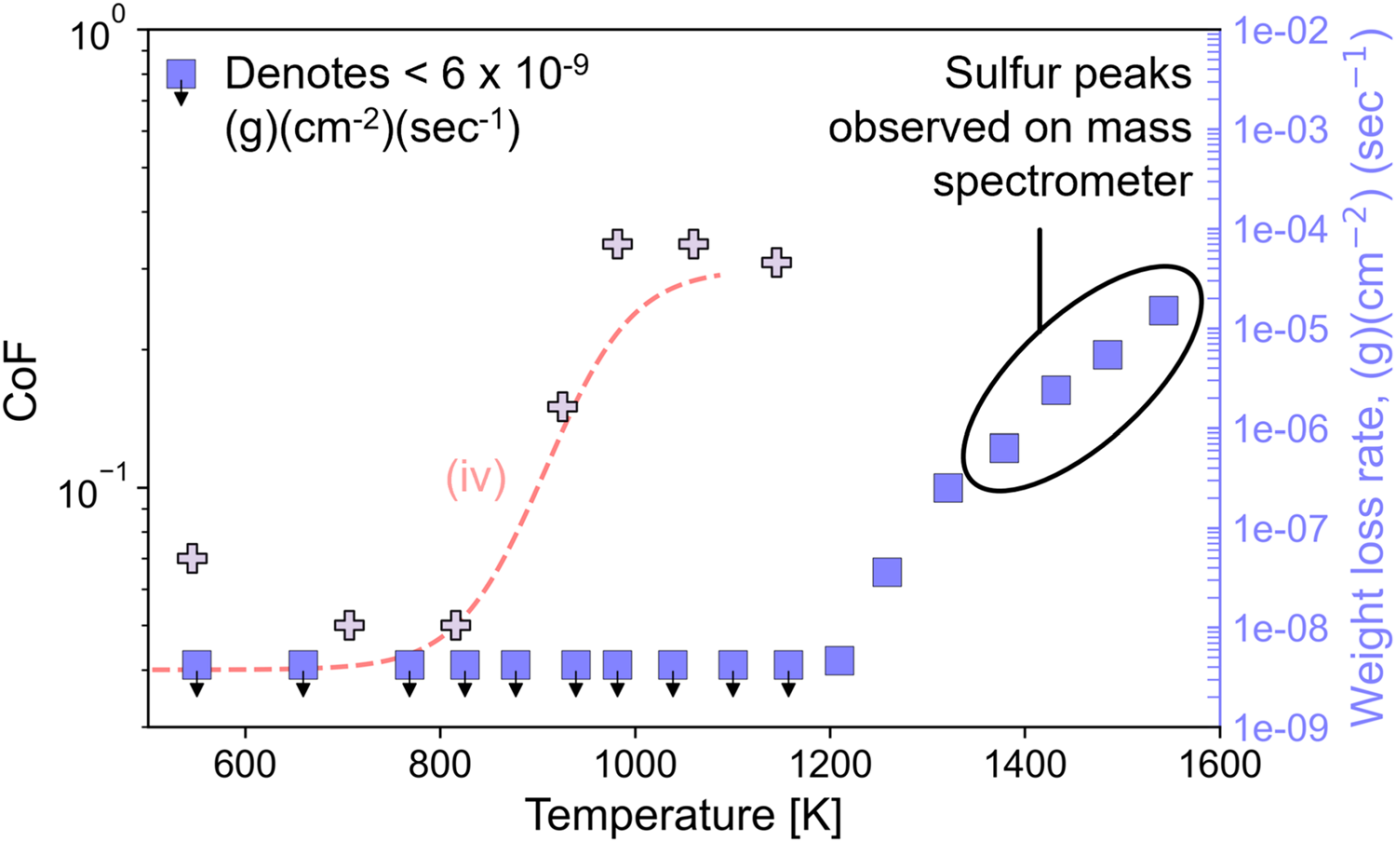
CoF from tribotests (plus symbols, left y-axis) and weight loss rate from thermal stability tests (blue square symbols, right y-axis) as a function of temperature. Trendline (iv) is the same as in Fig. 6. Sulfur was detected from mass spectroscopy at temperatures above 1350 K, but weight loss rate increased above 1203 K. The increase in CoF with increasing temperature occurred near 800 K, before the increase of weight loss rate and before sulfur was detected. Data replotted from [1] (Color figure online)

Trendline (iv) shows low friction above 540 K until a sharp increase after 800 K, supported by [1]. To explain that behavior, the authors performed thermal stability tests for MoS_2_ in vacuum between 500 and 1300 K in which stability was quantified by the intensity of the sulfur peak detected by a mass spectrometer and the reduction of weight of the MoS_2_ specimen. The results showed that MoS_2_ was thermally stable below 1203 K and then became less stable above that temperature. The data from the tribotests and stability tests are shown together in Fig. 7. The increase in friction occurred at a lower temperature than the increase in weight loss rate or observation of sulfur peaks in the spectrometer. This indicated that dissociation with resultant sulfur emission was unlikely to explain the high friction and accelerated wear at temperatures above 800 K [1]. Although not suggested by the authors, a possible explanation for the increased friction and wear may have been loss of cohesion within the burnished particle coatings.

## Friction and SWR Above 270 K in Air

### Identification of Trends Above 270 K in Air

**Fig. 8:**
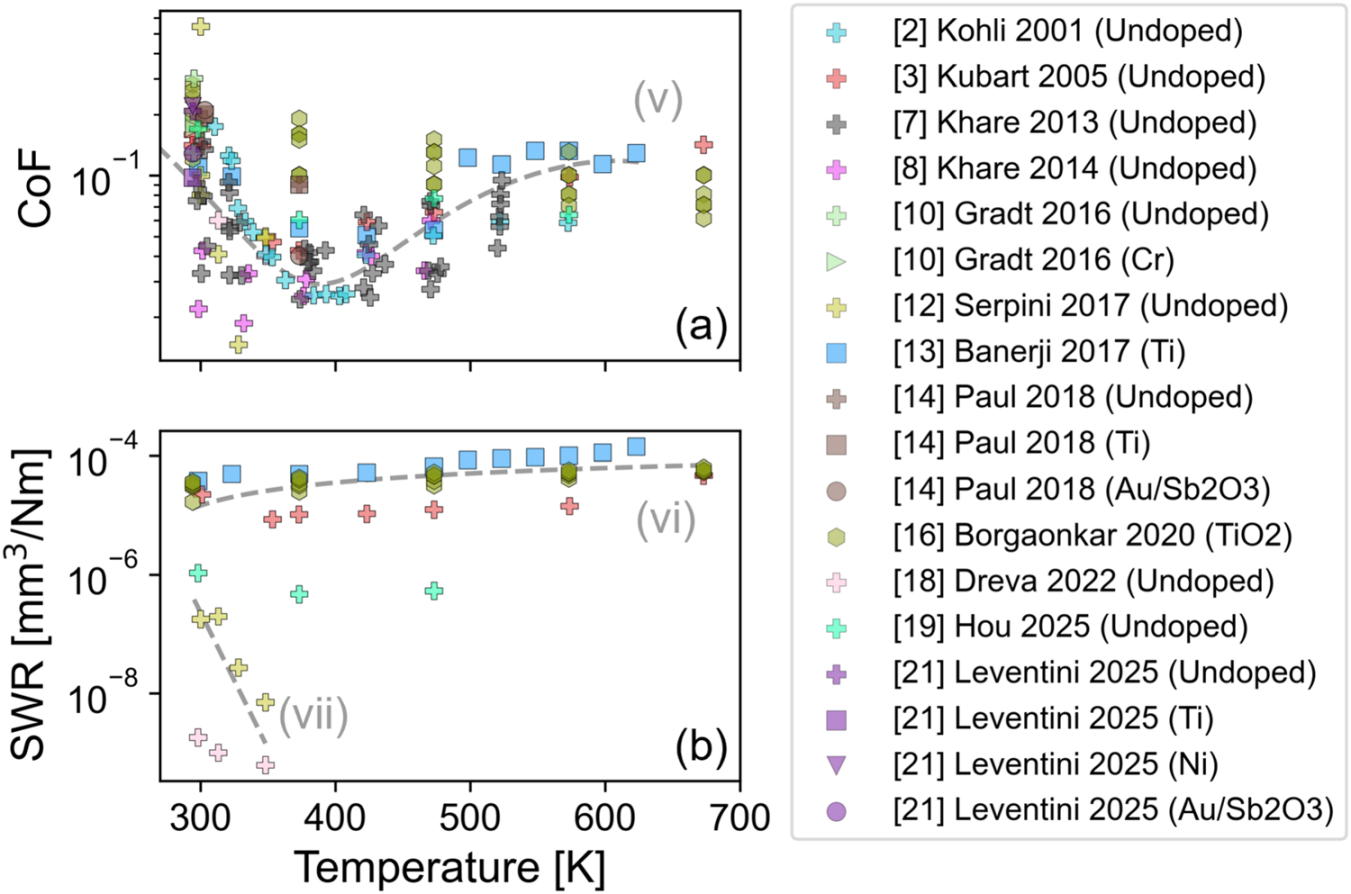
Focused view of **a** CoF and **b** SWR measured in air at temperatures between 270 and 700 K. The y-axis of **a** and **b** are presented on a logarithmic scale. Trendlines (v)–(vii) are included as a visual guide only. Trendline (v) shows a non-monotonic CoF trend with a minimum CoF around 373 K. Trendline (vi) shows an increase in SWR with increasing temperature while trendline (vii) shows a decrease in SWR with increasing temperature

Figure 8a shows the CoF of MoS_2_-based DFLs from tribotests performed at temperatures above 270 K in air. In these tests, the scatter in the data can be attributed to differences in material and test conditions, like in the vacuum and inert gas tests, but also to differences in relative humidity between the studies. However, taken together, the studies show that CoF decreases with temperature from room temperature to around 373 K and then begins to increase with further temperature increase, as highlighted by trendline (v).

This non-monotonic trend can be attributed to the combined effects of water and oxygen on coating performance. These effects were differentiated in studies by [7, 8] that performed pin-on-flat reciprocating tests under controlled environments. Reference [7] observed that, at temperatures below ~ 373 K, the presence of water significantly degraded performance while oxygen had little effect. At temperatures above ~ 373 K, oxygen caused an increase in the CoF while water (without oxygen) did not change the coating performance significantly. These conclusions underly the trends seen by most other studies (mechanisms discussed in the next section).

Between 270 and 373 K, References [2, 3, 7, 8, 12–14, 18] reported a decrease in CoF with increasing temperature in air. However, at higher temperatures, particularly above 470 K, some studies [2, 3, 7, 8, 13] reported that the CoF increased with increasing temperature. These studies used burnished MoS_2_ [2], sputter deposited pure MoS_2_ [3, 7, 8] and Ti-doped MoS_2_ [13]. Reference [2] performed tests in open air with no humidity control. Reference [3] performed ball-on-disk tribometer experiments in both dry nitrogen and humid air (35% and 50% RH). The study observed that, at 373 K, coating performance in the humid air environment was comparable to coating performance in dry nitrogen at room temperature (in agreement with [7] that showed that the influence of water waned above 373 K). Reference [13] performed tests in humid (45% RH) air conditions. The study reported a steep increase in CoF above 473 K and that the coating became ineffective above 673 K. Only one study, Reference [16], reported a contradictory trend in which CoF decreased with temperature up to 670 K in tests on burnished and cured MoS_2_/TiO_2_ composite (70 ± 5% RH).

The SWR under humid/dry air data shown in Fig. 8b can be separated into two groups, differentiated by two dashed trend lines. Trendline (vi) highlights studies that reported an increase in SWR with temperature from 270 to 700 K. This trendline is supported by [3, 13, 16]. Reference [3] observed a slow increase from 373 to 673 K and then, above 673 K, the SWR increased rapidly and the coating failed almost instantaneously in 50% RH. Reference [13] used burnished MoS_2_ based coatings and Reference [16] used sodium silicate-bonded, burnished MoS_2_ coatings. Both reported an increase in SWR as temperature increased in 45% and 70 ± 5% RH, respectively. For these two studies, the increase of SWR between 300 and 670 K was on the order of 1 × 10^−5^ mm^3^/Nm.

The second trendline (vii) shows a decrease in the SWR between 270 and 350 K. This trend is supported by [12, 18, 19], all of which involved coatings deposited by sputtering. Reference [12] measured the SWR during both ramp up and ramp down between room temperature and 348 K. The study observed that, at 348 K, the DFL had two orders of magnitude smaller SWR compared to at room temperature. Reference [18] observed that the SWR reduced by half as temperature increased from 298 to 348 K. Reference [19] performed tests in humid air (~ 65 RH%), observing a decrease in SWR between 298 and 373 K. The study also observed a slight increase between 373 and 473 K. However, at 573 K, SWR was not measured because the coating failed before the test finished, indicating the wear life had been reached.

### Mechanisms Proposed in Literature Above 270 K in Air

**Fig. 9:**
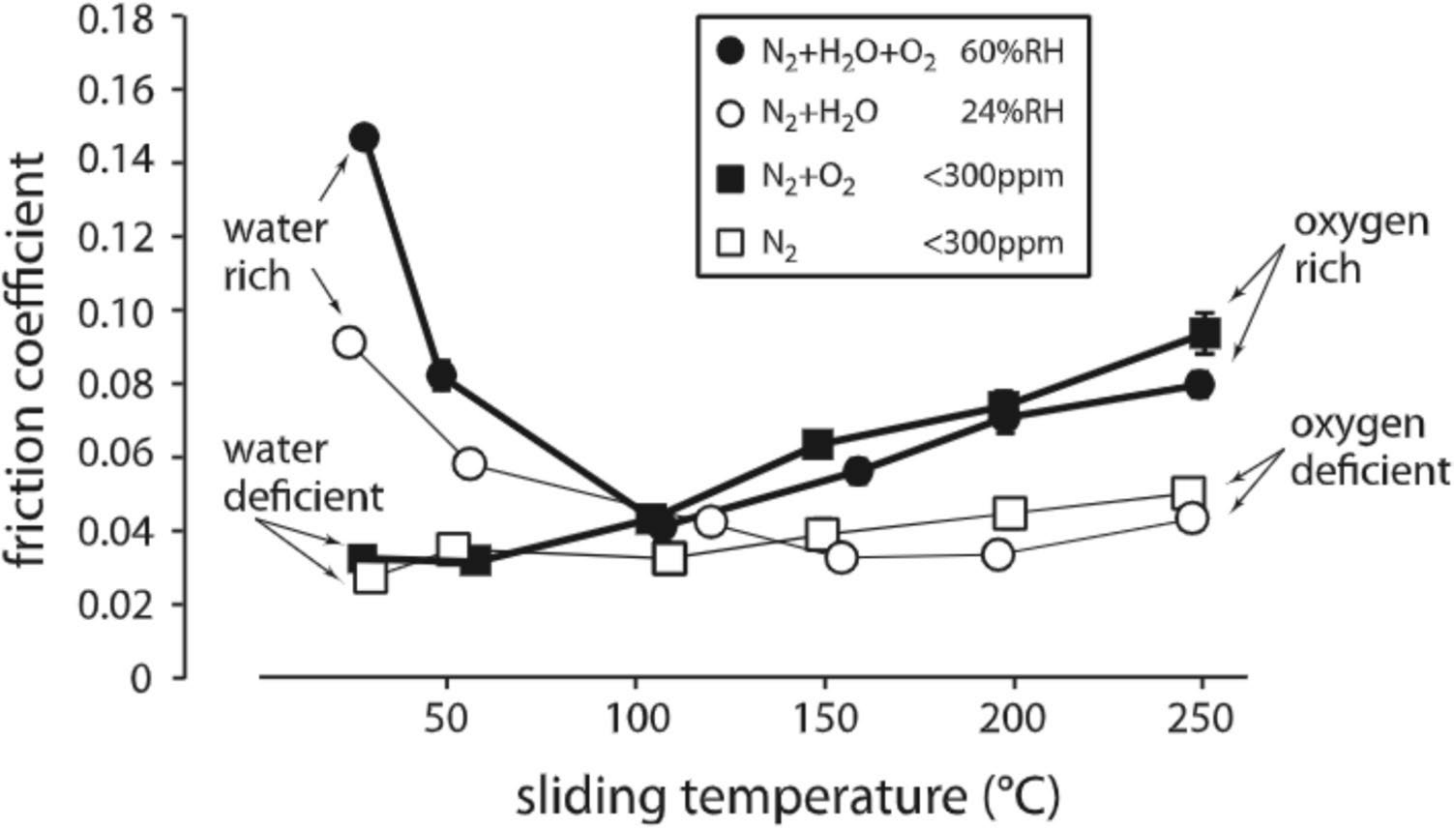
Friction in dry nitrogen, dry air, humid nitrogen, and humid air as a function of temperature. At low temperatures, water increases friction with or without oxygen while, at high temperatures, oxygen increases friction with or without water. Reproduced with permission [7]

The tribological behavior of MoS_2_-based DFLs above room temperature in humid and dry air environments is affected by two key mechanisms, water adsorption and oxidation. Particularly, DFL performance is determined by water and water adsorption at lower temperatures while performance is determined by oxygen and oxidation at higher temperatures. Reference [7] isolated the effects of water and oxygen identified the temperature at which the dominant factor changed from water to oxygen to be 373 K. The results of tests in water/oxygen rich/deficient environments that identified this transition temperature are shown in Fig. 9.

**Fig. 10:**
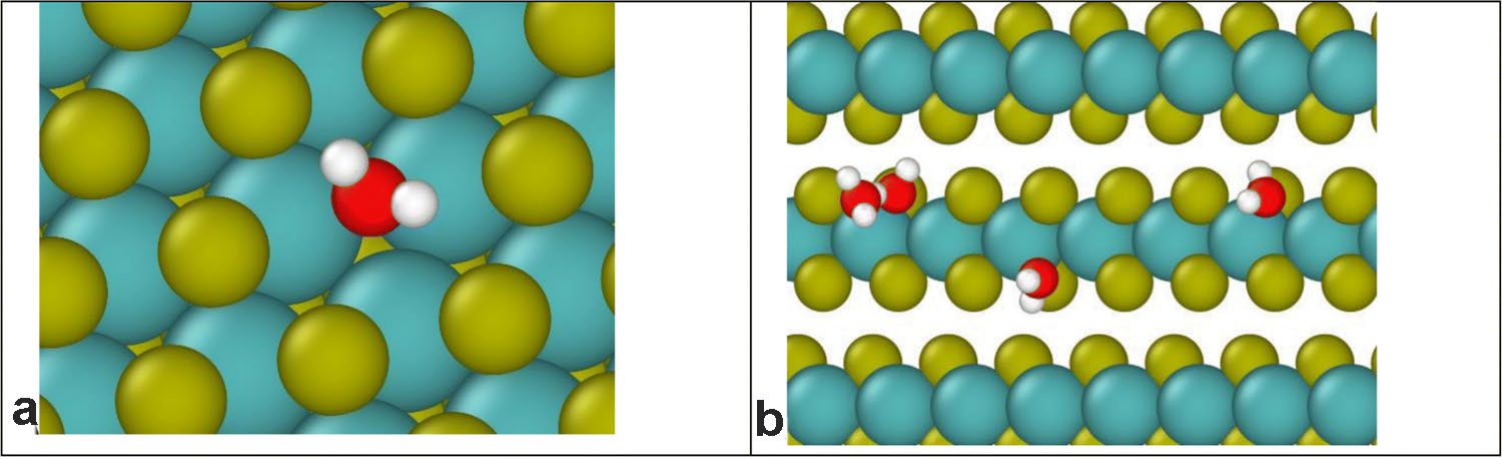
Snapshots from grand canonical Monte Carlo simulations of adsorption of water on MoS_2_ at either **a** defects on the basal surface or **b** defects at edges. Colors: yellow S, red O, white H, teal Mo. Reproduced with permission [42] (Color figure online)

At temperatures between 300 and 373 K, studies reported a reduction in CoF with increasing temperature and attributed that trend to the thermal desorption of water molecules from the MoS_2_ surface [8, 12, 18]. Simulations have shown that defects on surfaces or edges of lamellae strongly attract water molecules, as shown in Fig. 10. Physiosorbed water molecules hinder the alignment and easy shear of lamellae, increasing friction at room temperature. Temperatures between 300 and 373 K provide sufficient thermal energy to desorb water, promoting better lamellar alignment and reducing shear strength, while maintaining the composition and structure of MoS_2_. Reference [8] demonstrated that annealing MoS_2_ in humid environments at high temperatures effectively removed water adsorbed on the coating, reducing friction and wear. Similarly, Reference [18] observed that water desorption at temperatures up to 348 K facilitated the formation of basally oriented lamellae, lowering CoF and wear. Reference [12] corroborated these findings through Raman spectroscopy of wear scars noting that residual water vapor near the contact zone could increase the CoF during run-in. Moderate heating is beneficial because it facilitates the desorption of water. Reference [12] also found that the rate of desorption depends on humidity, with drier conditions promoting more efficient removal of any residual water on the coating and improved tribological performance.

**Fig. 11:**
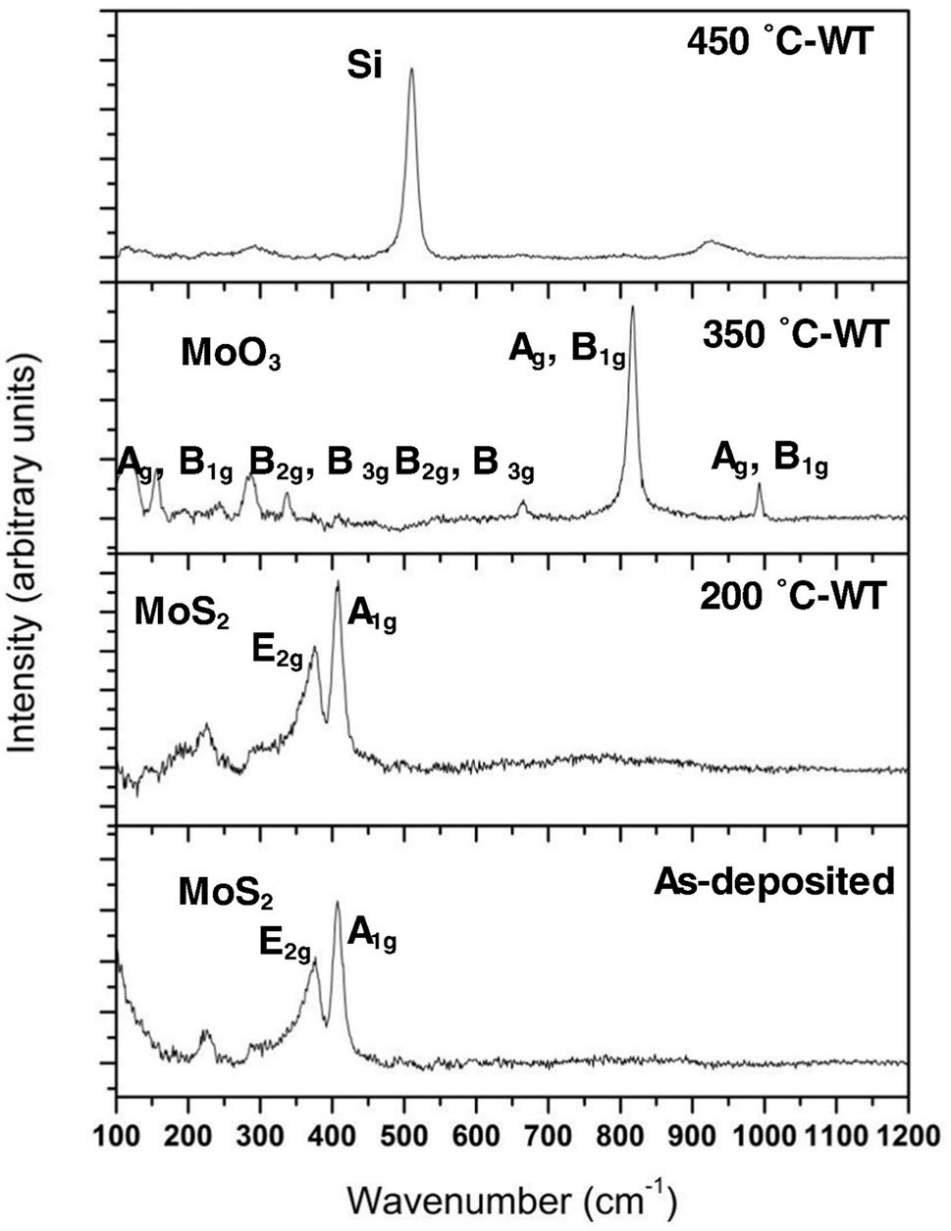
Raman spectra of as-deposited Ti-doped MoS_2_ and the wear tracks formed on the coating during tribotesting at three temperatures. At 623 K (350 °C in figure) the Raman peaks correspond to MoO_3_. Reproduced with permission [13]

At temperatures above 373 K, oxidation becomes the dominant factor for the behavior of MoS_2_. Oxidation leads to the formation of MoO_3_, which has higher interlayer adhesion and abrasive properties than MoS_2_ [43, 44]. This transformation results in increased friction and wear during sliding. Reference [13] observed that Ti-doped MoS_2_ coatings maintained low CoF and wear up to 473 K in 45% RH due to the formation of a MoS_2_-rich transfer layer. However, above 473 K, Raman spectroscopy measurements showed that coating oxidation increased significantly, leading to the formation of MoO_3_, as shown in Fig. 11. This change corresponded to a rise in CoF from 0.13 at 623 K to 0.34 at 773 K. Reference [18] noted that oxidation below 373 K in 35–50% RH had minimal impact on friction but, above this threshold, its effects became pronounced. Reference [2] identified a transition point at 393 K, where CoF, initially stabilized by water desorption, began to rise due to the onset of oxidation. Reference [7] showed that oxidation at higher temperatures is due to O_2_, whether or not H_2_O is present. The incorporation of dopants, such as Ti, Au, or Cr, has been shown to improve the oxidation resistance of MoS_2_ coatings. Reference [10] observed that Cr-doped MoS_2_ exhibited lower friction and SWRs in humid air (50% RH) compared to undoped MoS_2_ at elevated temperatures. Nonetheless, the overall trend of increasing CoF with temperature above 473 K persisted, indicating that, while dopants can slow oxidation, they cannot prevent it.

An interesting hysteresis phenomenon driven by both water and oxygen was observed from tests with heating and cooling cycles. Reference [7] found that, in high relative humidity environments, the presence of water appeared to mitigate oxidation of the coating by displacing oxygen or preferentially adsorbing onto the MoS_2_ surface. This resulted in reduced frictional hysteresis, with narrower hysteresis loops observed in humid conditions than in dry air. Reference [8] demonstrated that hysteresis behavior above 273 K was caused by oxidation of the coating. These studies showed that, at lower temperatures, high humidity adversely affects DFL performance due to water adsorption while, at higher temperatures, high humidity can be beneficial because water impedes oxidation.

## Gaps and Opportunities

In this review, we compiled and summarized previously reported temperature-dependent friction, SWR, and wear life trends for MoS_2_-based DFLs. Due to the challenges associated with measuring these parameters across a wide range of temperatures, most data sets from individual studies were relatively limited in scope. However, taken together, the results from many studies reveal generally consistent trends. These trends have been attributed to various mechanisms, sometimes only via suggestion and sometimes with evidence or indirect support. Bringing the data and proposed mechanisms together as we have done here reveals opportunities for the research community to more comprehensively characterize MoS_2_-based DFL temperature dependence and understand the underlying mechanisms.

### Limited Data

From Fig. 1, it is evident that there are some conditions where very little data is available.

There is generally less data, particularly SWR, below room temperature than above it. This is likely because heating is often simpler experimentally than cooling. Testing below room temperature requires very low relative humidity, which is typically achieved through a vacuum or inert gas atmosphere, to prevent condensation or freezing of water on the coatings. However, especially given the use of DFLs in space, cold temperature conditions are extremely important. Further, while most space applications will be in vacuum, there may be missions that require operation in cold with water in the atmosphere; for example, the icy moon Europa which is believed to have a subsurface ocean.

At very high temperatures (over 540 K), there is only one paper reporting friction data [1], and this was for burnished coatings which are rarely used in spacecraft. From the tribotests in that study, the DFL failed around 800 K, but the thermal stability tests revealed that dissociation was not observed until around 1350 K. This higher dissociation temperature of MoS_2_ is supported by another study in which high-resolution photoelectron spectroscopy showed pristine Mo and S peaks with the 2:1 ratio and low-energy electron diffraction pattern showed good surface crystallinity up to 1125 K [45]. The lower temperature failure in [1] might have been caused by loss of cohesion between particles in the burnished coatings, possibly due to desorption of chemical species that enhance interparticle adhesion. This hypothesis could be evaluated by conducting controlled water and/or hydrocarbon adsorption measurements along with temperature cycling during tribotesting. Such studies would be relevant to the high temperatures that may occur in some space applications, such as the sun-facing side of the International Space Station, missions to Venus, and missions near the sun.

Figure 1 clearly shows there are only a few studies that report wear life in any condition. This is because wear life tests can run for a long time (days or even weeks), especially in vacuum/inert gas conditions, which limits the total number of tests that can be run for a given coating and condition. However, for DFLs that will be used in space, wear life is extremely important since these coatings cannot be repaired or replaced once they leave earth, so coating life determines application life [46]. Therefore, it would be beneficial if more wear life data could be collected as a function of temperature.

Generally, more data collected across a wider range of temperatures and at different test conditions is needed. This will be a challenge for the typical approach of measuring tribological data one test at a time. However, recent developments have enabled high throughput measurements for MoS_2_ DFLs [28], and these could be leveraged to comprehensively characterize the temperature-dependence of the DFLs rapidly and consistently.

### Conflicting Trends

Although many trends are consistent across the various studies reviewed here, there are also contradictions.

Some studies performed tests with temperature cycling and reported inconsistent behavior. In Reference [11], multiple temperature cycles were performed in low temperature GN_2_ environment, resulting in CoF values that varied significantly in between cycles at comparable temperatures specially around 220 K, as shown in Fig. 5. However, Reference [9] also performed temperature cycle tests and did not observe significant hysteresis. This difference may be explained by the fact that Reference [9] tested undoped MoS_2_ while Reference [11] tested Au/Sb_2_O_3_-doped MoS_2_, suggesting the hysteresis in [11] could have been caused by the increased expression of the dopant species. It remains unclear whether other doped-MoS_2_ DFLs would exhibit the same hysteresis behavior. Additionally, it is possible that residual water contaminants, methods of cooling/heating, or environment (vacuum vs. inert gas), contribute to hysteresis observed below 273 K, but further investigation is needed.

In Fig. 8a, the CoF measured in air at higher temperatures, particularly above 470 K, was reported to increase with increasing temperature by most studies [2, 3, 7, 8, 13]. In contrast, Reference [16] reported that CoF decreased with temperature up to 670 K. This discrepancy could be due to differences between the tests in [16] and the other studies, including that the DFL in [16] was a silicate-bonded MoS_2_/TiO_2_ coating applied to the substrate by burnishing (as opposed to sputtering) and the humidity was higher (70 ± 5% RH) than the others.

The SWR below 270 K in vacuum/inert gas environment has not been widely studied. Results from the two studies that did report SWR under these environments at different temperatures [6, 11] are not in agreement. Reference [6] reported lower SWR at colder temperatures and attributed this to a higher probability of forming smaller MoS_2_ flakes at lower temperatures. In contrast, Reference [11] reported higher SWR at colder temperatures. They attributed this opposite trend to the same flake size mechanism, supported by the SEM measurement of a decrease in average particle size from 2 µm at room temperature to ~ 500 nm at 173 K. The detrimental effect of smaller flakes was supported by two simulation-based studies. Reference [20] showed that smaller flakes hinder the formation of basally oriented layers and Reference [15] showed that smaller flakes have a higher energy barrier to sliding. The effect of cold temperature on SWR as well as the underlying mechanism require further study.

In air tests above room temperature, the SWR was reported to either increase or decrease with increasing temperature, highlighted by the two different trendlines in Fig. 8b. The increasing trend was supported by [13, 16] while the decreasing trend was supported by [12, 18, 19]. The difference cannot be attributed to dopants since undoped DFLs were used for one or more studies exhibiting both trends. The humidity ranges were similar as well. One possible cause of the discrepancy could be differences in deposition parameters, which have been shown to affect the performance of MoS_2_-based DFLs [28]. Further research is needed to isolate the cause.

### Test Methods

Most tribotests used to study temperature-dependent MoS_2_ DFL behavior were performed using similar tribometer configurations and test parameters. However, these configurations and parameters may not reflect the operating conditions of some MoS_2_ DFL applications.

All data reported here was collected using tribotests with pure sliding motion. While pure sliding tribotests may capture the most severe conditions possible within a rolling element bearing, they do not mimic the typical or expected operation. For example, sliding contact is not consistent with the rolling motion of rolling element bearings that operate at very low slide-to-roll ratios. One previous study reported the tribological performance of MoS_2_-based DFLs in rolling between coated and uncoated cylinders [47]. Other MoS_2_ studies have used a Spiral Orbit Tribometer [48–50] that enables rolling, sliding, and pivoting, reflecting motion experienced by a ball in an angular contact bearing. Performing such rolling contact tests at different temperatures would be very useful.

Most previous studies, and all those included in this review, involve point contact. This may not reflect the contact geometry of some aerospace components, such as gear systems where the contact between meshing gear teeth is a line, or ball bearings where the contact between the ball and inner and outer rings is a rolling ellipse. Non-circular contacts are characterized by larger contact sizes and lower contact pressures, which means that the performance of a MoS_2_ coating may differ when used to lubricate these different geometries. MoS_2_ DFLs have been tested with line contact in previous studies using block-on-ring [51–53], vee-block-on-pin [51], and ring-on-disc [39] tribotests. Performing tests with these geometries at different temperatures can confirm if the trends and behavior identified in this review are applicable to non-point contact.

To achieve low humidity conditions, more tests use inert gas than vacuum due to the relative simplicity of testing with inert gas. Inert gas is used as a proxy for vacuum to facilitate earth testing, but the environment does not fully replicate the ultra-low-pressure conditions of space. Especially, partial pressure from the gas can prevent contaminant outgassing effects, potentially skewing the influence of friction and wear mechanisms. Preheating samples before testing is one method expected to help drive off trace contaminants. However, there has been no direct comparison between vacuum and inert gas chambers focusing on the effect of preheating on temperature-dependent DFL performance.

### Material Properties

Some of the scatter in the data in Figs. 2, 6, and 8 is attributable to variations in substrate and coating material properties between the different studies.

It is well known that MoS_2_ DFL performance depends on substrate [29, 30] and counterbody material [54, 55]. The question is whether these materials affect temperature dependence. One material-related mechanism for temperature dependence is thermal mismatch between the substrate and coating. If this mechanism is dominant in a given condition, changing the substrate material may affect temperature dependence. Similarly, temperature dependence has been attributed to the effectiveness of the MoS_2_ film transferred to the counterbody, a process that is necessarily affected by the surface chemistry and material properties of the counterbody. Most tribotests focusing on temperature dependence have been performed with bearing steel substrates and counterbodies. However, since some space applications use ceramics or unconventional alloys such as Ti-6Al-4V, it is important for future studies to evaluate the temperature dependence of MoS_2_-based DFLs deposited on a wide range of potential substrate and counterbody materials.

The addition of dopants has been shown to affect MoS_2_ DFL temperature dependence [4, 9, 11, 14, 21]. Reference [4] evaluated five different doped DFLs and showed their temperature-dependent friction differed dramatically. The difference was attributed to the effect of the dopants on thermal activation energy. Reference [14] studied one undoped and two doped MoS_2_ coatings and showed that the coating CoF and wear rate was influenced by dopants. References [9, 11] proposed temperature-dependent friction and wear were affected by the effect of running film/transfer film stability on the expression of doping species (Au and Sb_2_O_3_) at the sliding surface. Reference [21] studied four different doped and undoped MoS_2_ coatings and reported that the effect of decreasing temperature on CoF and wear life of the coatings was affected by the composition of the coatings. Additional studies comparing the temperature dependence of different dopants as well as differing dopant concentrations in the coatings would inform selection of dopants for a given temperature range as well as a better understanding of how dopants affect temperature dependence.

Coating deposition method and parameters have been shown to affect MoS_2_ microstructure which in turn affects DFL performance [17, 25, 56]. A previous study demonstrated that the microstructure of MoS_2_ can influence its reactivity to oxidation [57]. Other studies have shown that microstructure determines the effectiveness of the running process during which basally oriented lamella form as well as the transfer film process [32–35]. Since microstructure is determined during coating deposition, correlating deposition method to temperature dependence would enable better control of this important parameter to optimize DFL performance.

### Mechanisms

Many different mechanisms have been proposed to explain the various trends observed. Some of these are widely cited, but still not fully understood or insufficient evidence is available to support them.

The CoF trends in Fig. 2a show a transition from lower to higher friction around 220 K, from data collected with different coatings and instruments across five different studies [4, 5, 9, 11, 17]. Most of these studies attribute the trend to thermal activation. However, a remaining question about this hypothesis is what physical or chemical process is being thermally activated. Particularly, Reference [5] was performed using AFM where the thermally activated stick–slip motion of the tip is between the AFM tip and MoS_2_ (assuming no wear). The other four studies were performed using ball-on-disk where, assuming a transfer film forms, relative motion (possibly through sticking and slipping) is between layers of MoS_2_. While either can be a thermally activated process, it is still not clear why the transition temperature is around 220 K for both. Another potential explanation for the transition temperature at 220 K is related to the possibility that water is trapped in the DFL and released during sliding. When the DFLs are tested in vacuum or inert gas, removal of MoS_2_ layers brings trapped water molecules to the new surface of the DFL where they can either disrupt the sliding process, as discussed previously, or desorb. Desorption is a temperature dependent process, so it is possible that the 220 K transition is related to the properties of water itself. Future work with experiments as well as simulations may focus on this aspect. Theoretical studies trying to harmonize these ideas such that the thermal activation mechanism can be applied across coatings, environments, and measurement methods would be beneficial.

The function of MoS_2_ as a DFL involves the transfer of some material from the coating to the counterbody, i.e., formation of a transfer film. Many previous studies have reported that the performance of the DFL is strongly determined by the effectiveness of the transfer film [11, 37–40]. However, the transfer film process is transient and occurs within the sliding contact. This means transfer films are difficult to characterize, which inhibits our understanding of the fundamental mechanisms behind them. Some studies have characterized the transfer film in post-test analysis using optical microscopy, X-ray diffraction, scanning electron microscopy, and other surface analytical instruments [38, 40, 58–60]. These have shown that the formation, chemical transformation, and microstructural evolution of the transfer film are critical in reducing friction and wear. Performing similar analyses of the transfer film as a function of temperature could be extremely useful in understanding the mechanisms of temperature dependence.

### Simulations

Most previous work on the temperature dependence of MoS_2_ DFLs has been performed using experimental methods. Tribometer measurements complemented by pre- and post-test surface characterization have provided evidence to support suggested mechanisms. However, as mentioned in the context of transfer film analysis, the key processes that underly DFL performance occur between two surfaces in relative motion where they are difficult to observe directly. This suggests that simulations could play an important role in our understanding of mechanisms. Previous simulation-based studies of MoS_2_ have been performed using first principle studies and MD simulations [15, 42, 61–77]. Such studies have helped us understand the mechanisms of water adsorption as well as oxidation of MoS_2_ [61, 75]. They also have provided information about the atomistic origins of the mechanical properties of MoS_2_ [62–64, 77].

Only a few MD simulation studies focused on temperature dependence of MoS_2_ [15, 20, 74]. Reference [20] suggested that MoS_2_ debris size is an important mechanism for temperature dependence. Reference [15] presented a model that helps correlate macroscopic shear strength of MoS_2_ to interlamellar translation of MoS_2_. Reference [74] performed temperature-dependent nanoindentation through MD simulations and computed the mechanical properties of MoS_2_ between 4.2 and 500 K. Further, existing modeling approaches primarily focus on pure MoS_2_, whereas most tribotesting and applications are with doped coatings. For MD simulations, this limitation arises primarily from the lack of appropriate force fields parameterized for doped MoS_2_. Only a few force fields for doped MoS_2_ exist but they have not been used to study temperature dependence [69, 71, 78]. Given that dopants significantly influence friction behavior across temperatures, future modeling strategies should incorporate dopant effects.

Beyond MD, there are a few papers that study the mechanical properties MoS_2_ for electronics applications using multi-scale simulation approaches [79, 80]. Multiscale simulations have also been used to model wear mechanisms for other tribological materials [81, 82]. Similar efforts can be made to model temperature-dependent MoS_2_ friction and wear mechanisms to capture the inherently multi-scale processes believed to affect DFL performance, e.g., lamella orientation and transfer film formation. Finally, the use of machine learning for MoS_2_ DFLs is relatively new [28] and can be leveraged, along with more experiment and simulation data, to enable prediction of temperature-dependent DFL performance.

## Conclusions

This review article summarized and analyzed previous studies that reported the friction, wear, or wear life of MoS_2_-based DFLs as a function of temperature. Trends reported in literature were not always consistent and multiple mechanisms have been proposed. However, a few general observations can be made based on the studies reviewed here. Testing in inert gas or vacuum at moderate temperatures (between 220 and 540 K) showed a relatively small effect of temperature on MoS_2_ DFL performance. Below 220 K, performance consistently deteriorated. This has been attributed to the adverse effects of cold on film microstructure, especially the formation of basally oriented lamella and effective transfer films; cold may also affect the expression of dopants. Below 220 K, thermally activated processes such as dislocation propagation may be slowed, which can adversely affect tribological performance. Above 540 K, limited data is available, but one study suggested loss of cohesion within the coating resulted in an increase in friction. Testing in air at temperatures above 270 K revealed a non-monotonic relationship between temperature and DFL performance and opposing trends were reported in the same temperature range. These observations have been explained by the competing effects of temperature on water desorption and oxidation. Particularly, increasing temperature increases desorption, which is beneficial to DFL performance, but it accelerates oxidation, which is detrimental. Both mechanisms are affected by the coating composition and the environment, leading to sometimes contradictory trends in literature. Although the comprehensive review performed here revealed these general trends, it also highlighted many opportunities for future work to fully characterize trends and understand the fundamental mechanisms.

Understanding the temperature-dependence of MoS_2_ DFL tribological behavior is extremely important for the effective use of MoS_2_ DFLs in MMAs that are critical to the success of space missions. The compilation of previously reported data in this review article will enable more accurate prediction of the performance of DFLs across the wide range of temperatures they may experience in space applications. Further, the analysis of mechanisms proposed to explain observed trends enables better understanding of MoS_2_ DFLs and may suggest avenues for optimizing their performance for a specific temperature range or environment through the design process. Lastly, the gaps and opportunities identified here can guide future studies that will complement the existing knowledge to enhance the reliability and performance of MoS_2_-based dry film lubricants in space environments.

## Supplementary Information

Below is the link to the electronic supplementary material.

Supplementary file1 (DOCX 2956 kb)

Supplementary file2 (CSV 56 kb)

## Data Availability

All data from literature used to make the plots in this review is provided in the supplementary information.
