# Supplementary material for: Temperature‑Dependent Friction, Wear, and Life of MoS₂ Dry Film Lubricants for Space Mechanisms: A Comprehensive Review
Source: Tribol Lett. 2025 Aug 1;73(4):114. doi: 10.1007/s11249-025-02052-6 (PMC12316733; doi:10.1007/s11249-025-02052-6)
Supplement: Supplementary file 1 — Supplementary file1 (DOCX 2956 kb) [file 11249_2025_2052_MOESM1_ESM.docx]

**Supplementary Information**

**Temperature Dependence of MoS_2_ Dry Film Lubricants: A Review**

Abrar Faiyad^1+^, Daniel Miliate^1+^, Samuel Leventini^1^, Jeffrey R. Lince^2^, Ashlie Martini^1*^

1. University of California Merced, Department of Mechanical Engineering

2. Space Tribology Consulting, Inc.

+ Co-first authors

* Corresponding author: [amartini@ucmerced.edu](mailto:amartini@ucmerced.edu)


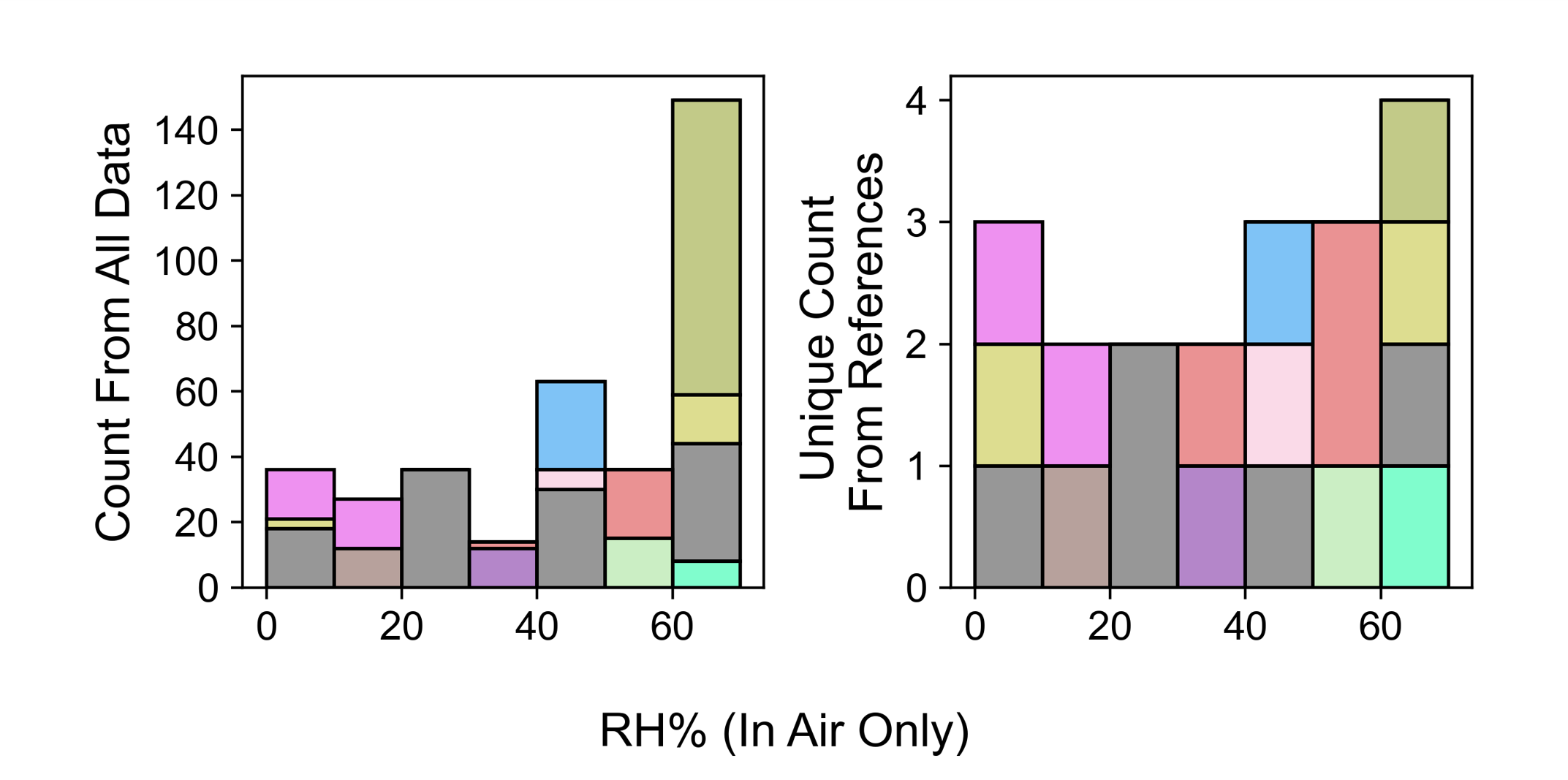


Figure S1: A stacked distribution of RH% for (left) the total count and (right) count by reference (i.e., one count per paper per bin). Colors represent the reference, defined in the legend of Figure 1.


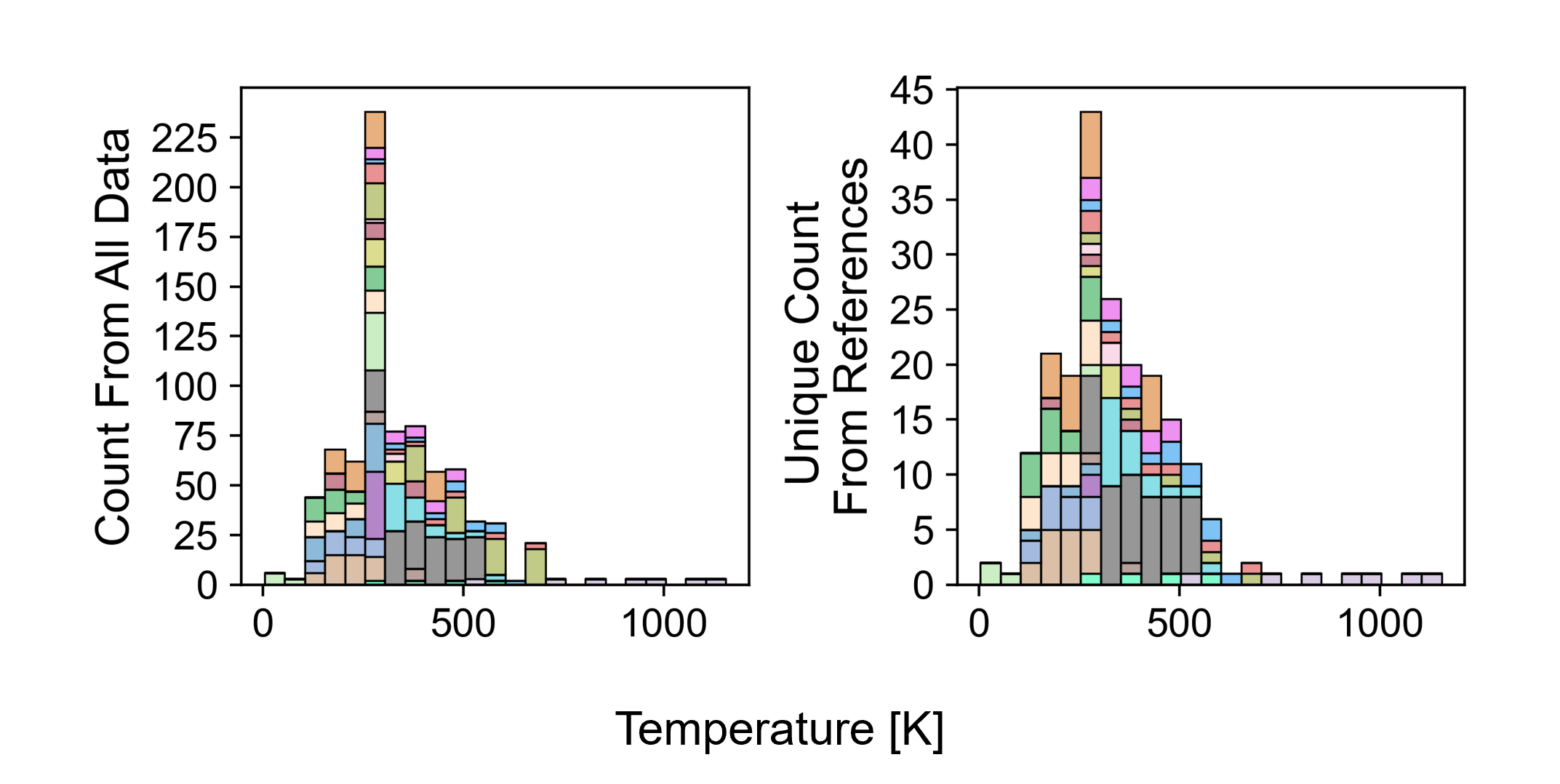


Figure S2: A stacked distribution of temperature for (left) the total count and (right) count by reference (i.e., one count per paper per bin). Colors represent the reference, defined in the legend of Figure 1.


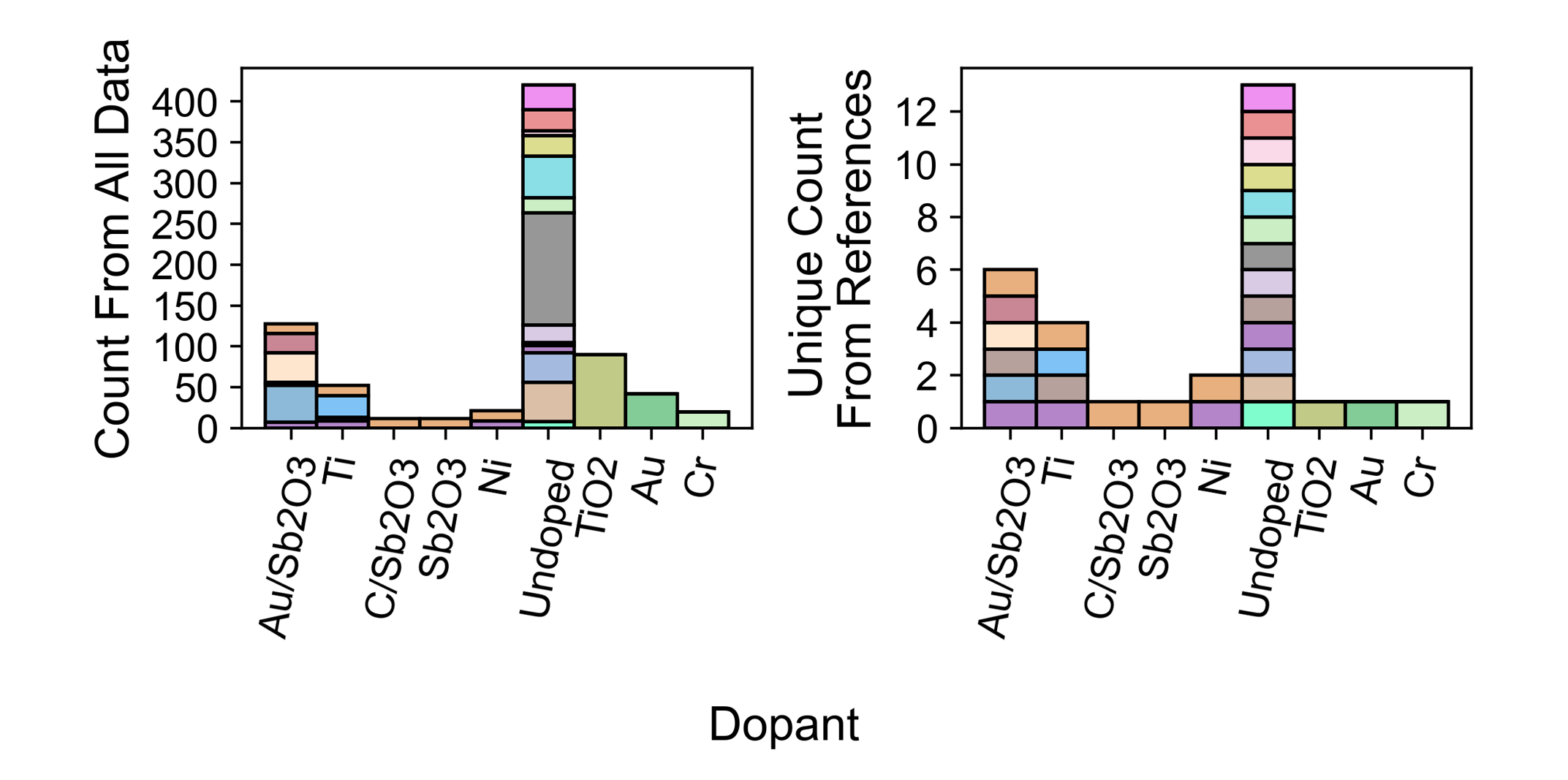


Figure S3: A stacked distribution of dopants, if any, for (left) the total count and (right) count by reference (i.e., one count per paper per bin). Colors represent the reference, defined in the legend of Figure 1.


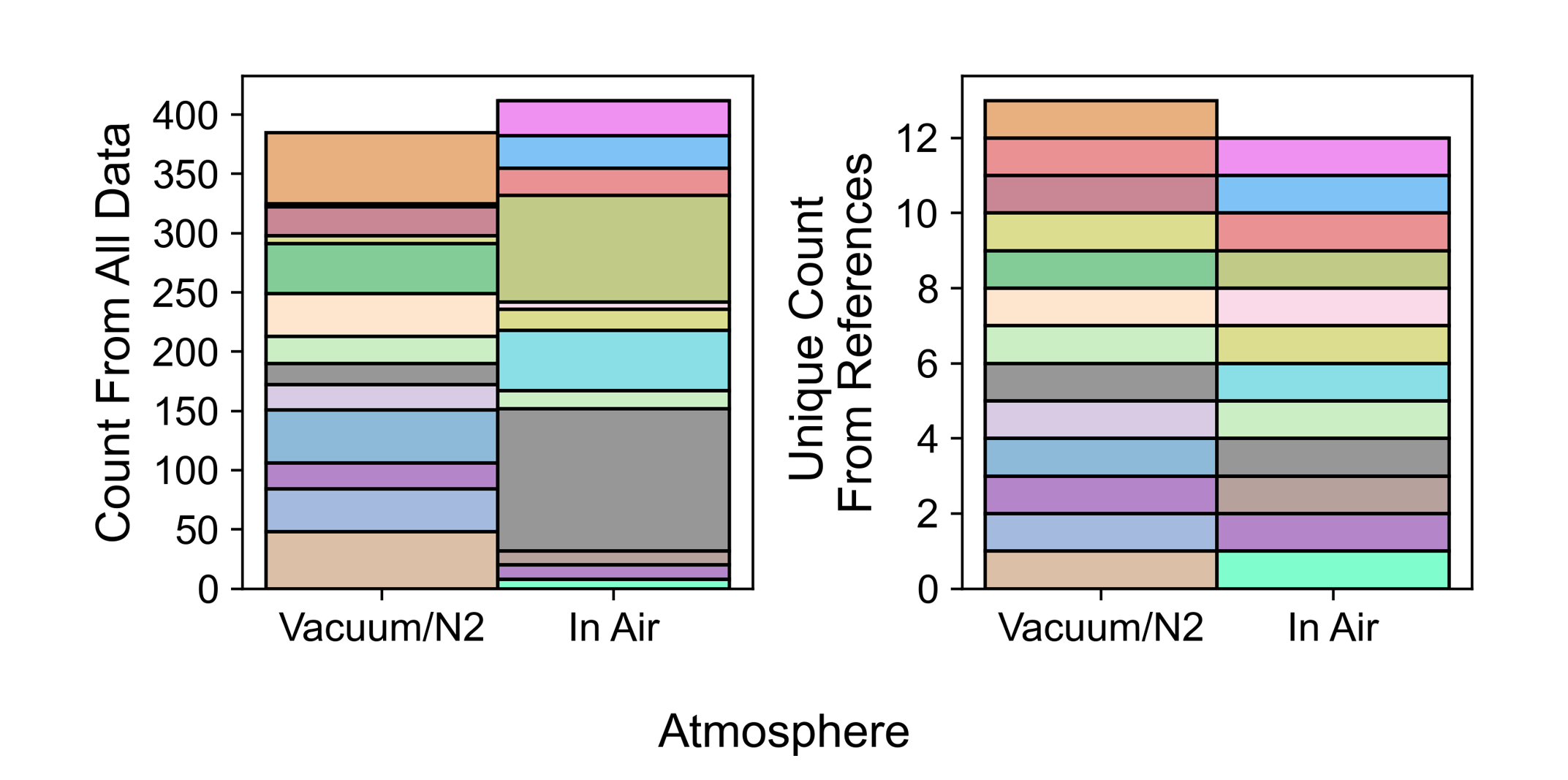


Figure S4: A stacked distribution of testing environment for (left) the total count and (right) count by reference (i.e., one count per paper per bin). Colors represent the reference, defined in the legend of Figure 1.


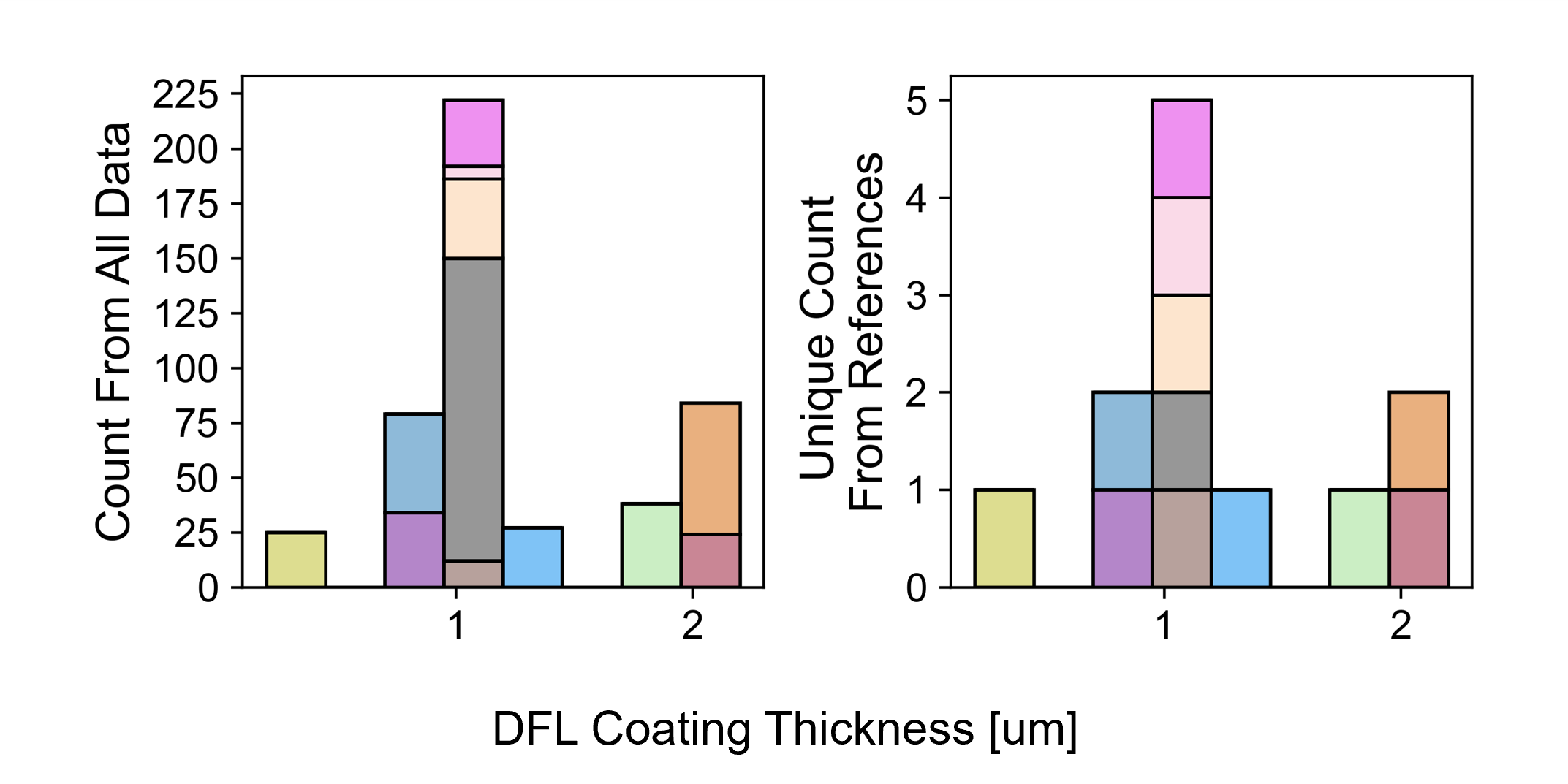


Figure S5: A stacked distribution of MoS_2_-based DFL coating thickness for (left) the total count and (right) count by reference (i.e., one count per paper per bin). Colors represent the reference, defined in the legend of Figure 1.


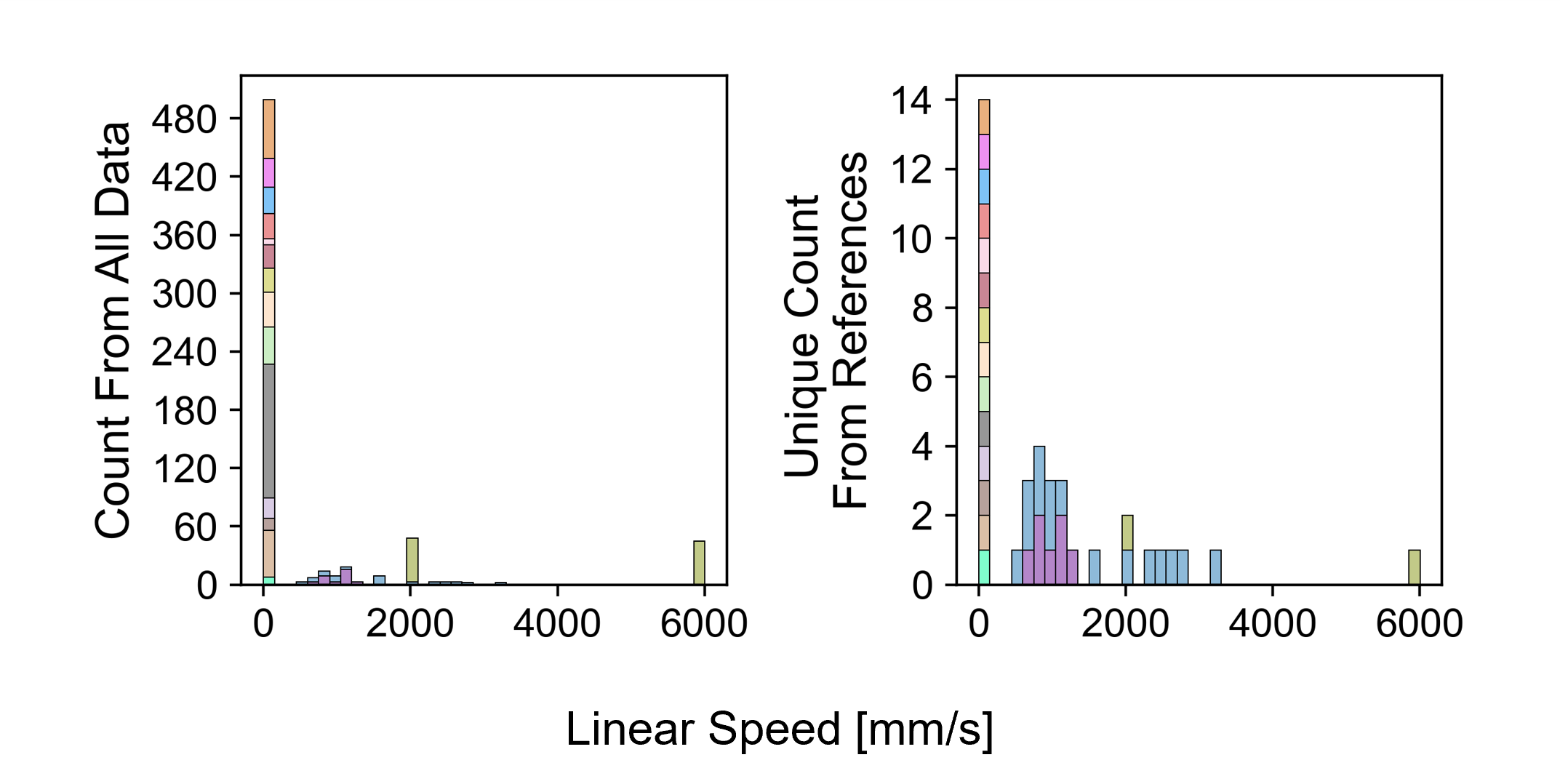


Figure S6: A stacked distribution of linear speed for (left) the total count and (right) count by reference (i.e., one count per paper per bin). Colors represent the reference, defined in the legend of Figure 1.


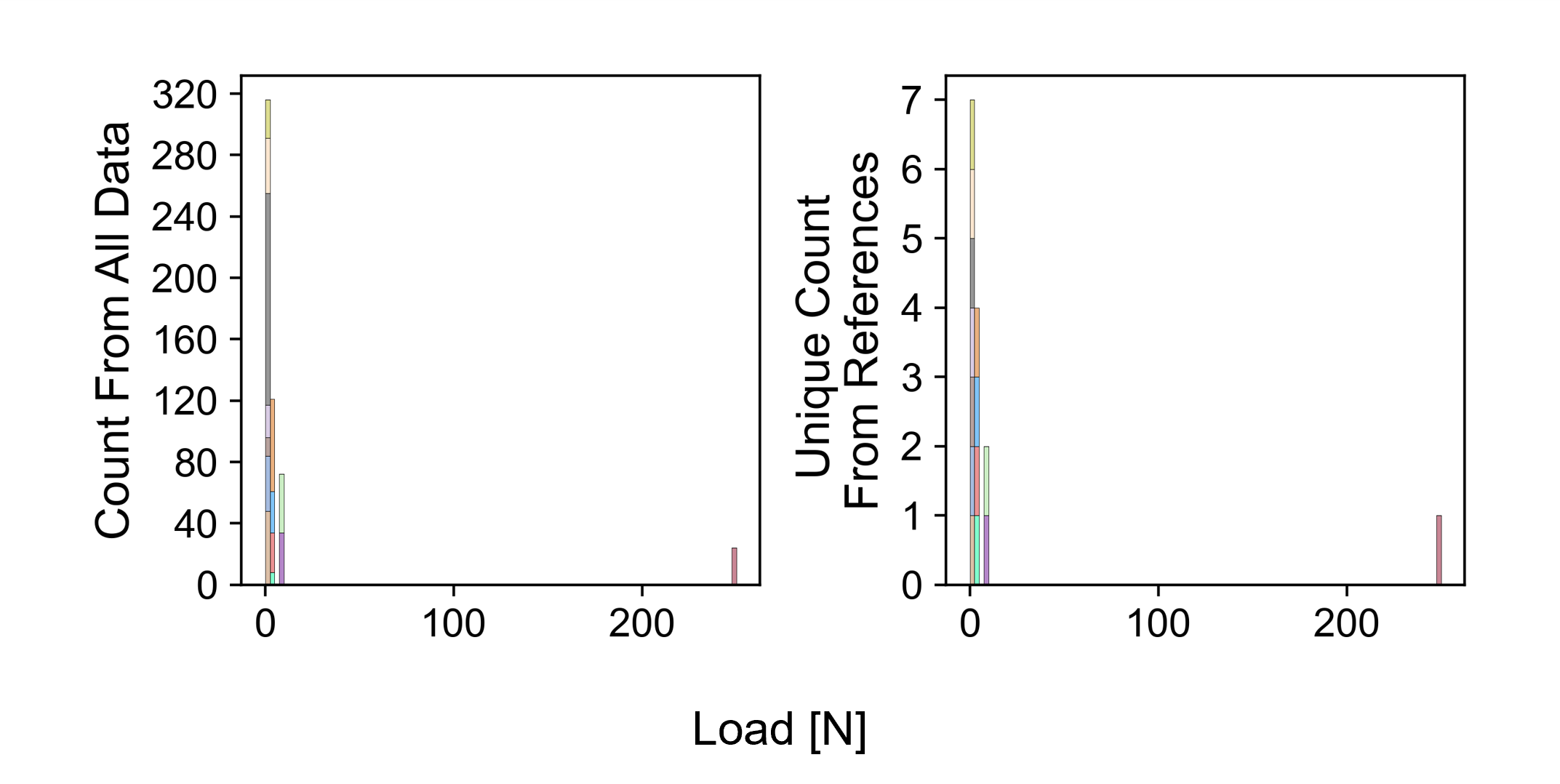


Figure S7: A stacked distribution of load for (left) the total count and (right) count by reference (i.e., one count per paper per bin). Colors represent the reference, defined in the legend of Figure 1.


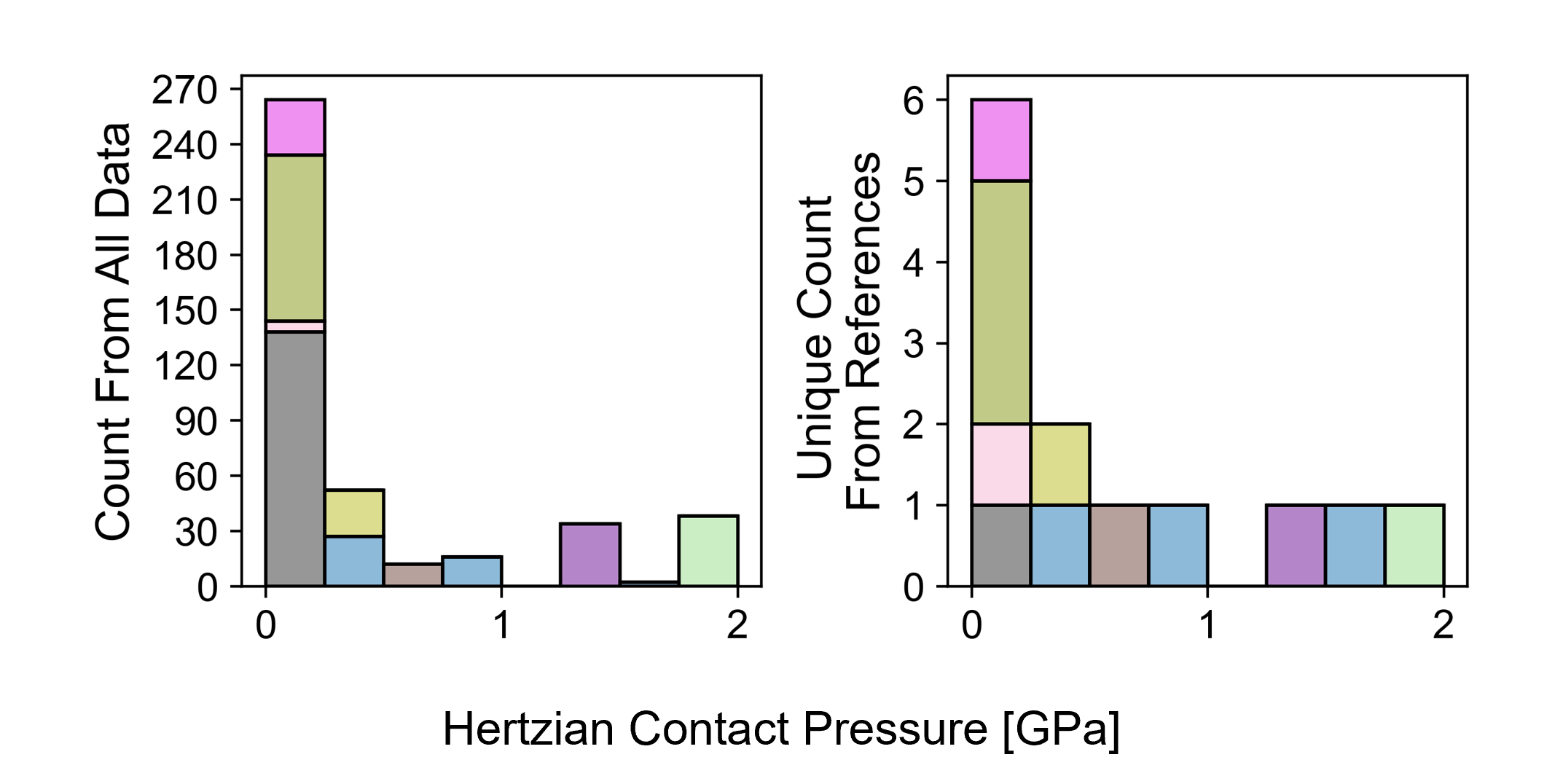


Figure S8: A stacked distribution of Hertzian contact pressure for (left) the total count and (right) count by reference (i.e., one count per paper per bin). Colors represent the reference, defined in the legend of Figure 1.


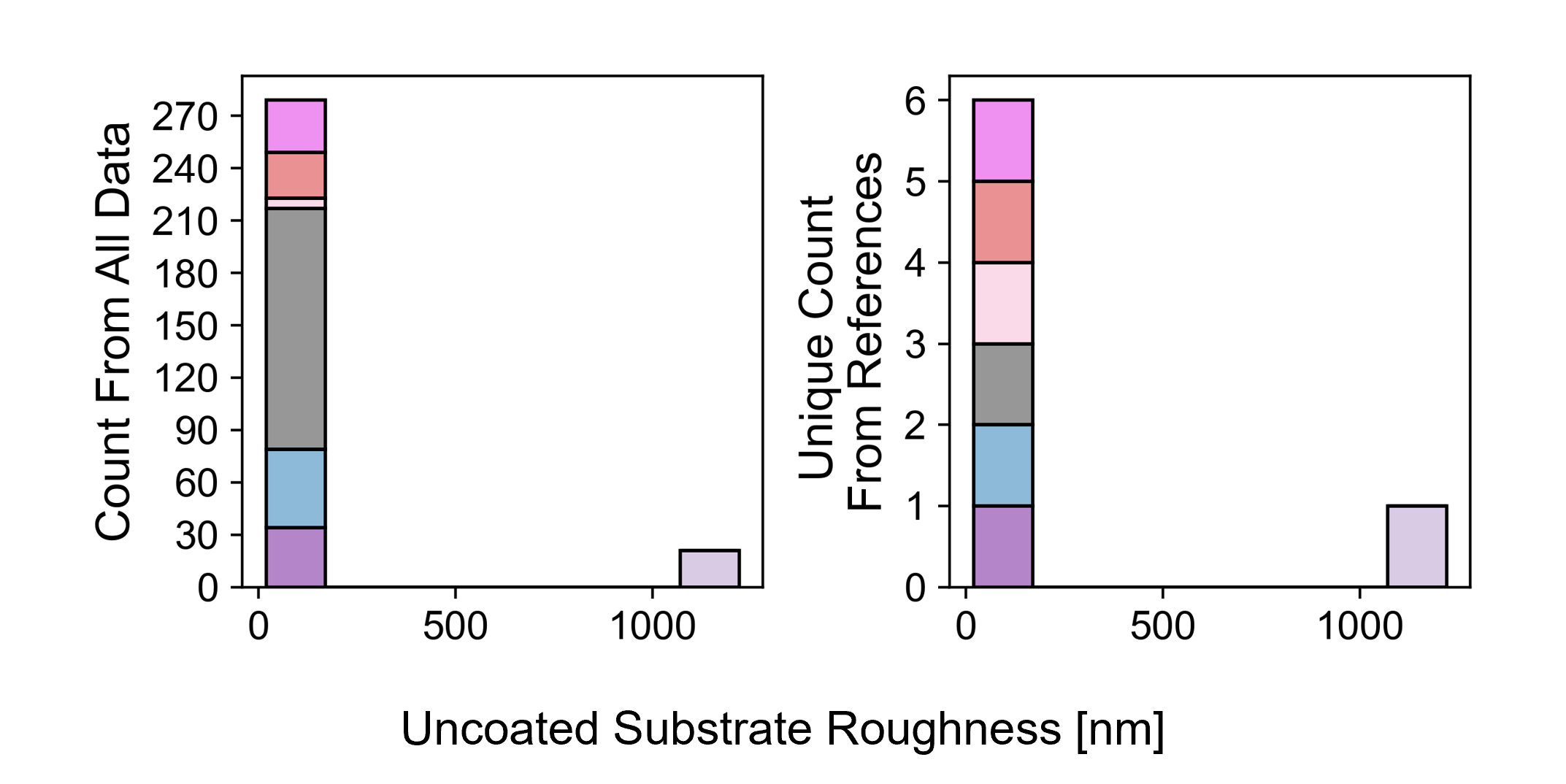


Figure S9: A stacked distribution of uncoated substrate roughness for (left) the total count and (right) count by reference (i.e., one count per paper per bin). Colors represent the reference, defined in the legend of Figure 1.


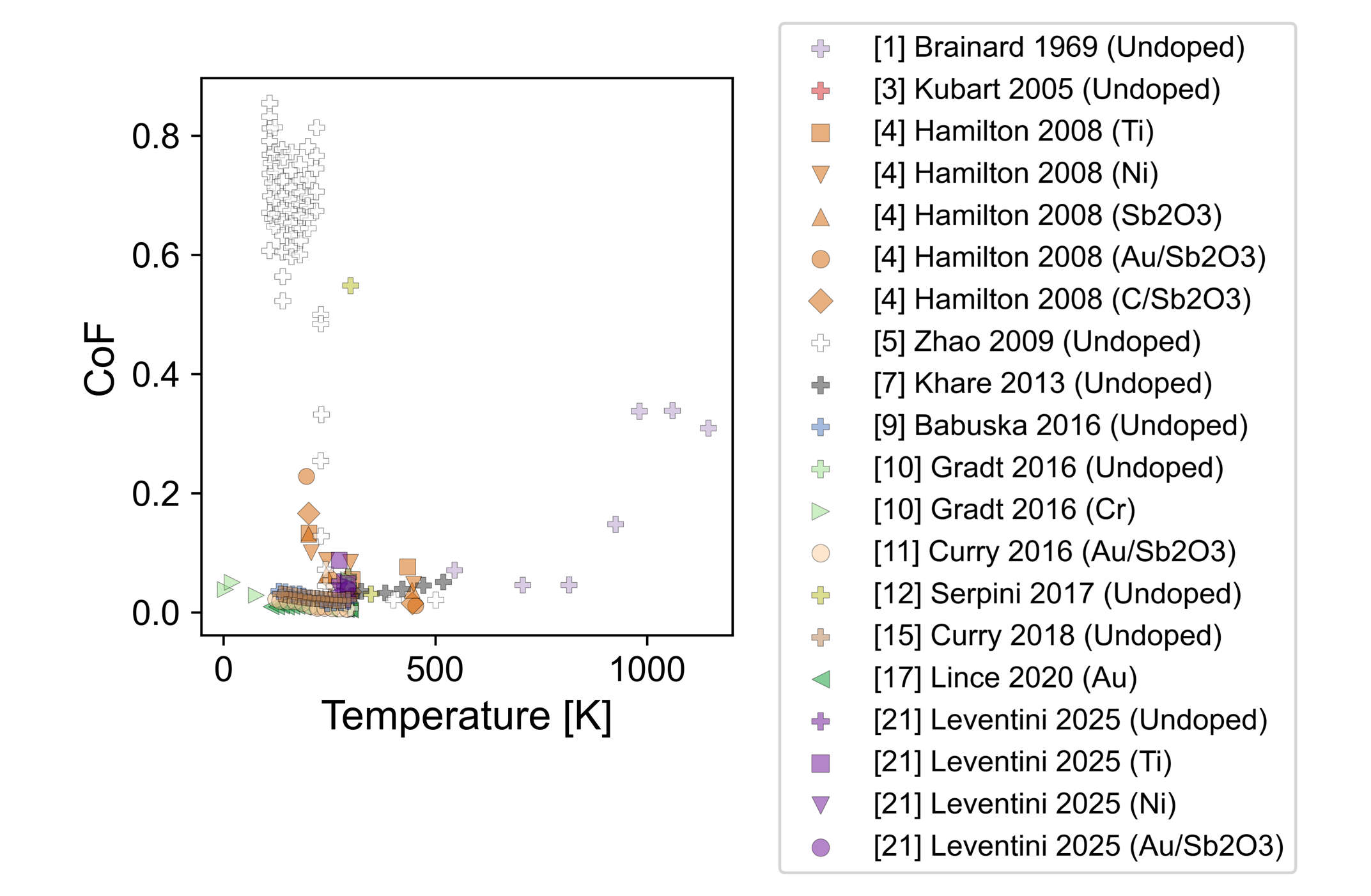


Figure S10: A focused view of results from vacuum or inert gas conditions between 0 and 1200 K, showing CoF


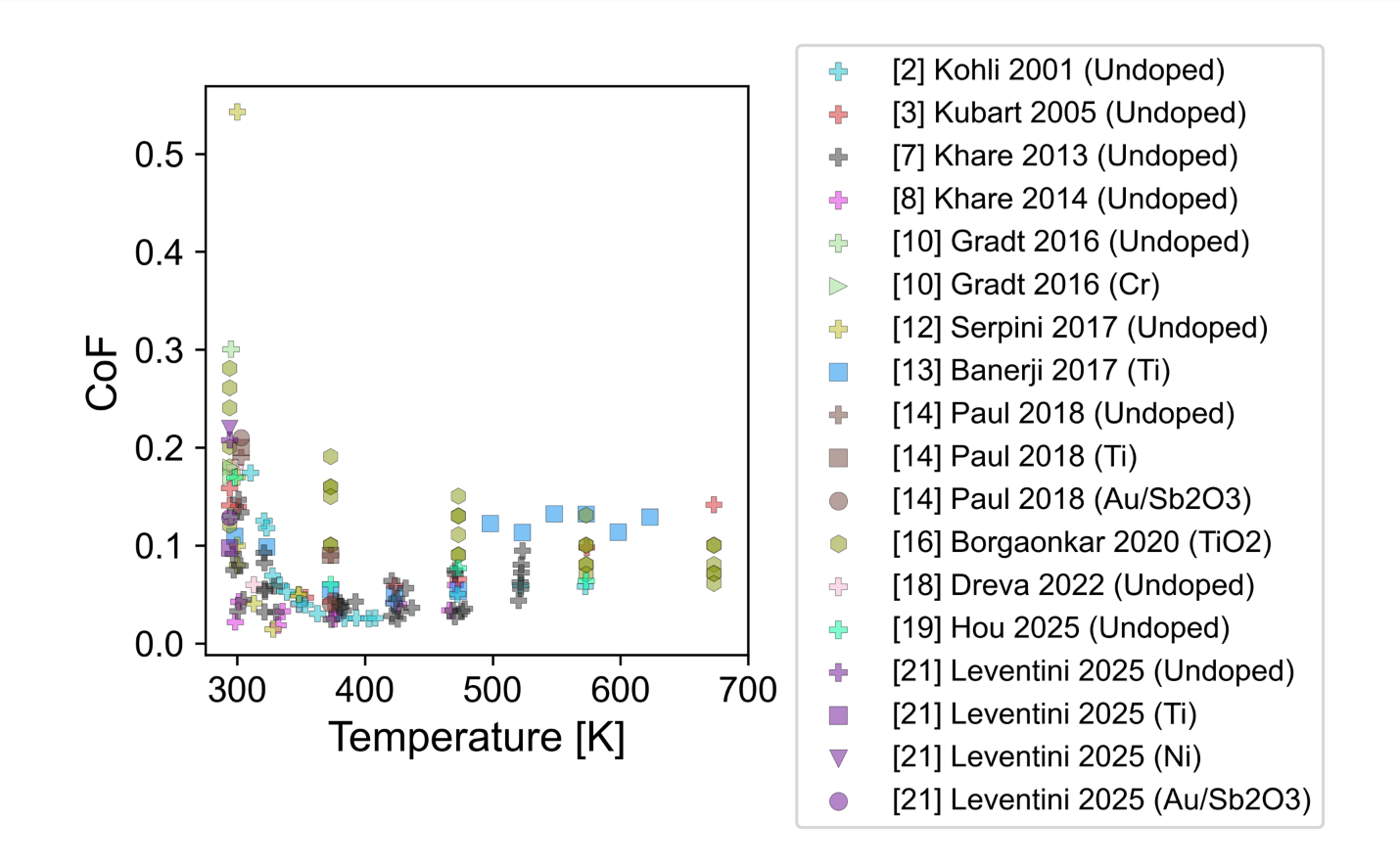


Figure S11: Focused view of CoF measured in air at temperatures between 270 and 700 K.


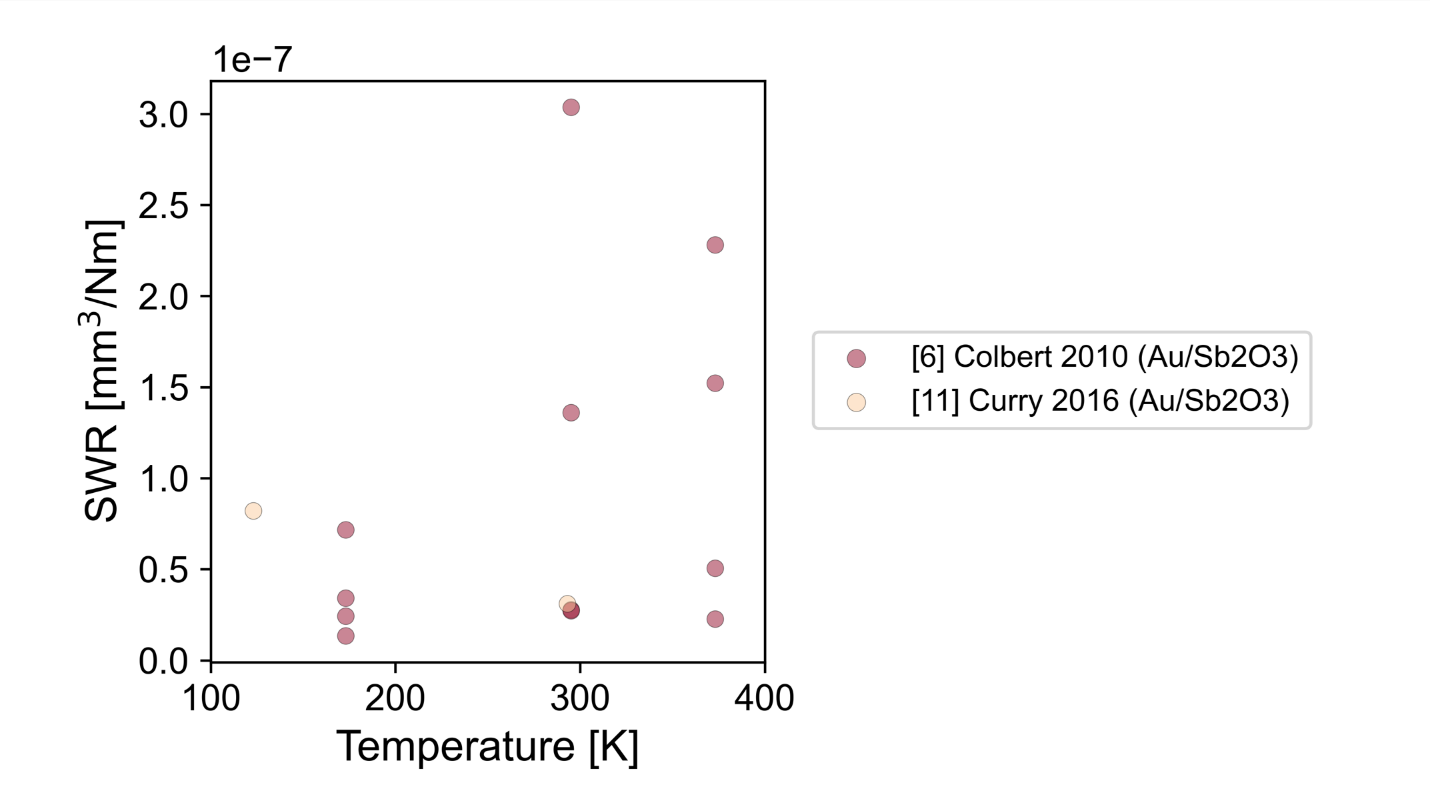


Figure S12: A focused view of results from vacuum or inert gas conditions below 400 K showing SWR


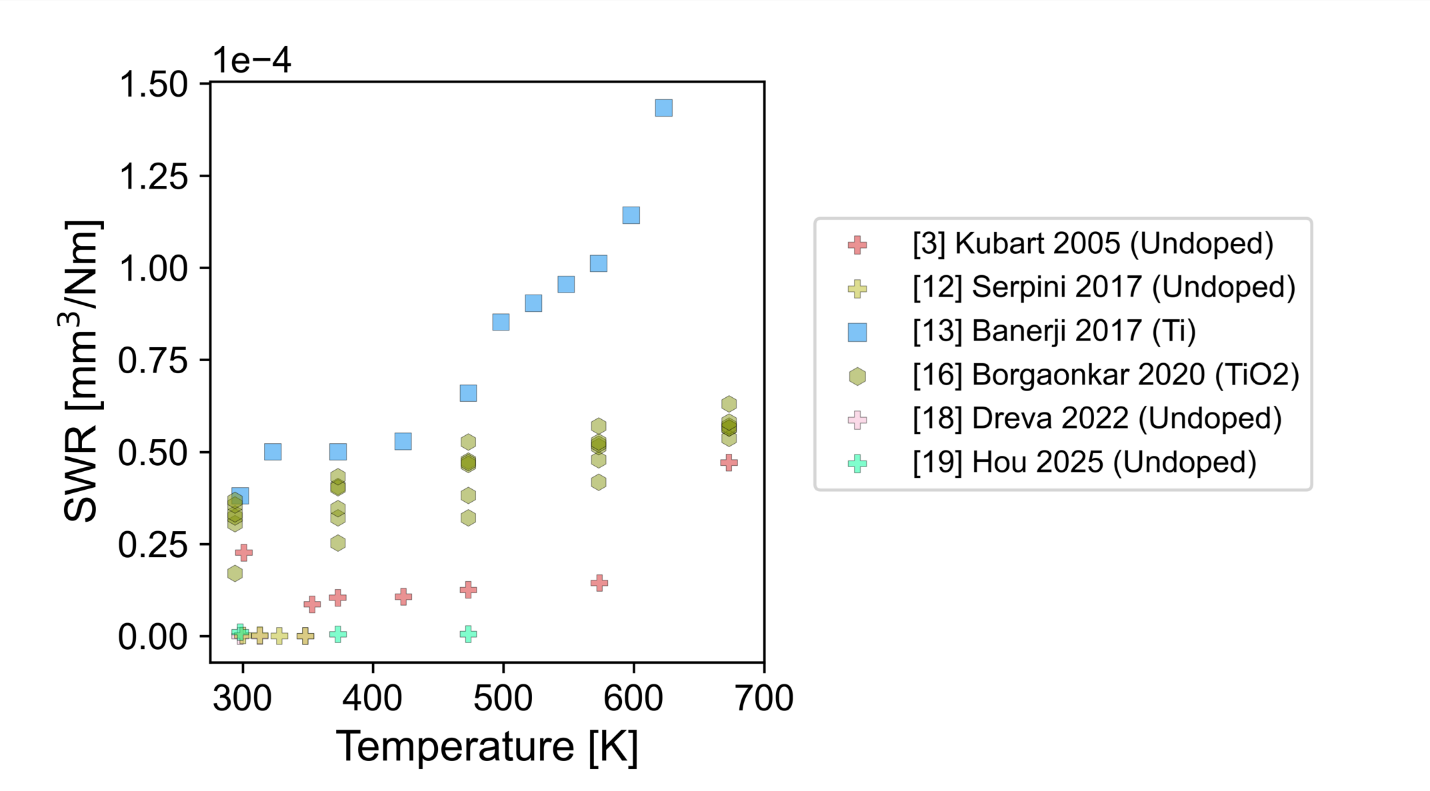


Figure S13: A focused view of results from air conditions below 700 K, showing SWR.


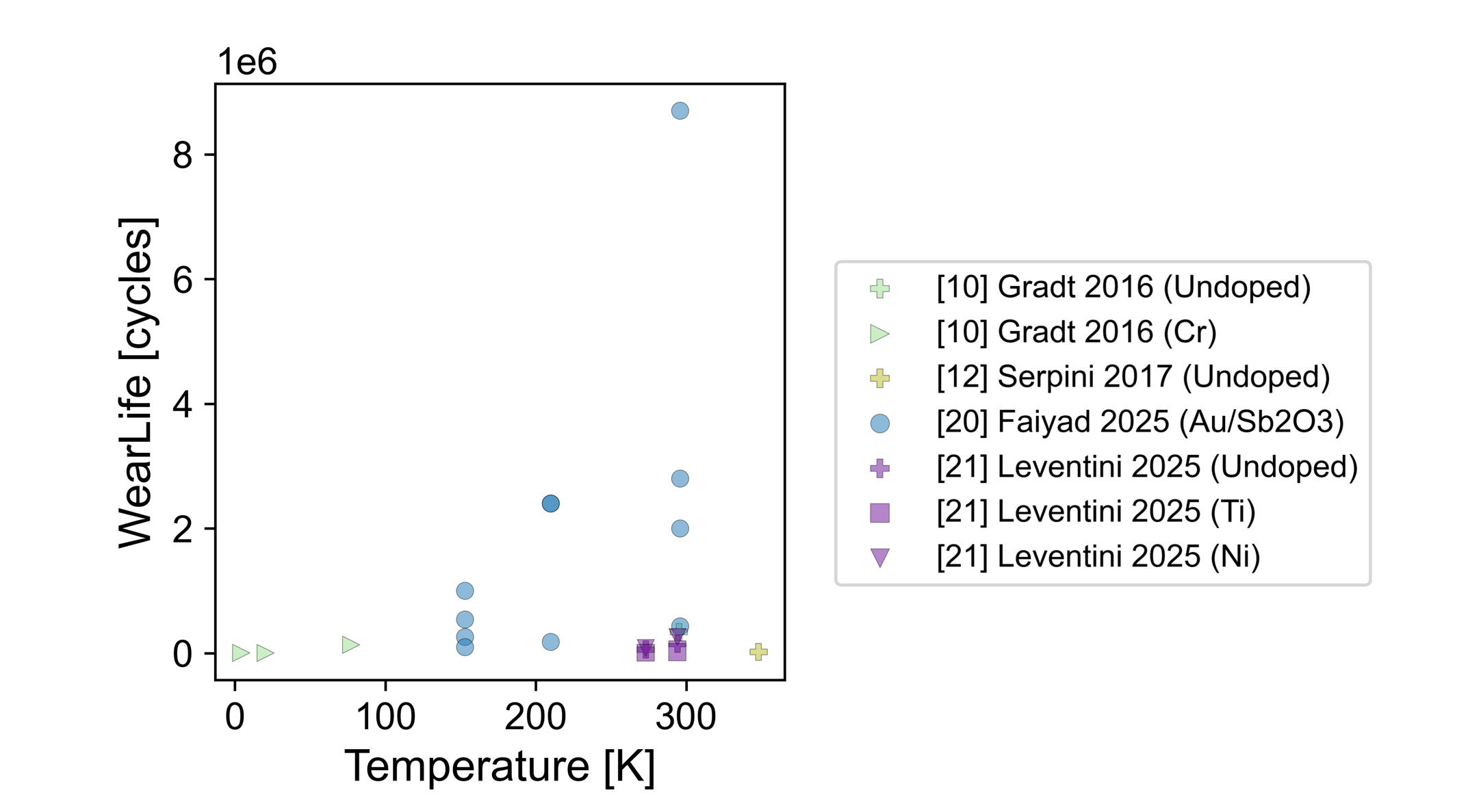


Figure S14: A focused view of results from vacuum and inert gas conditions below 400 K, showing wear life.


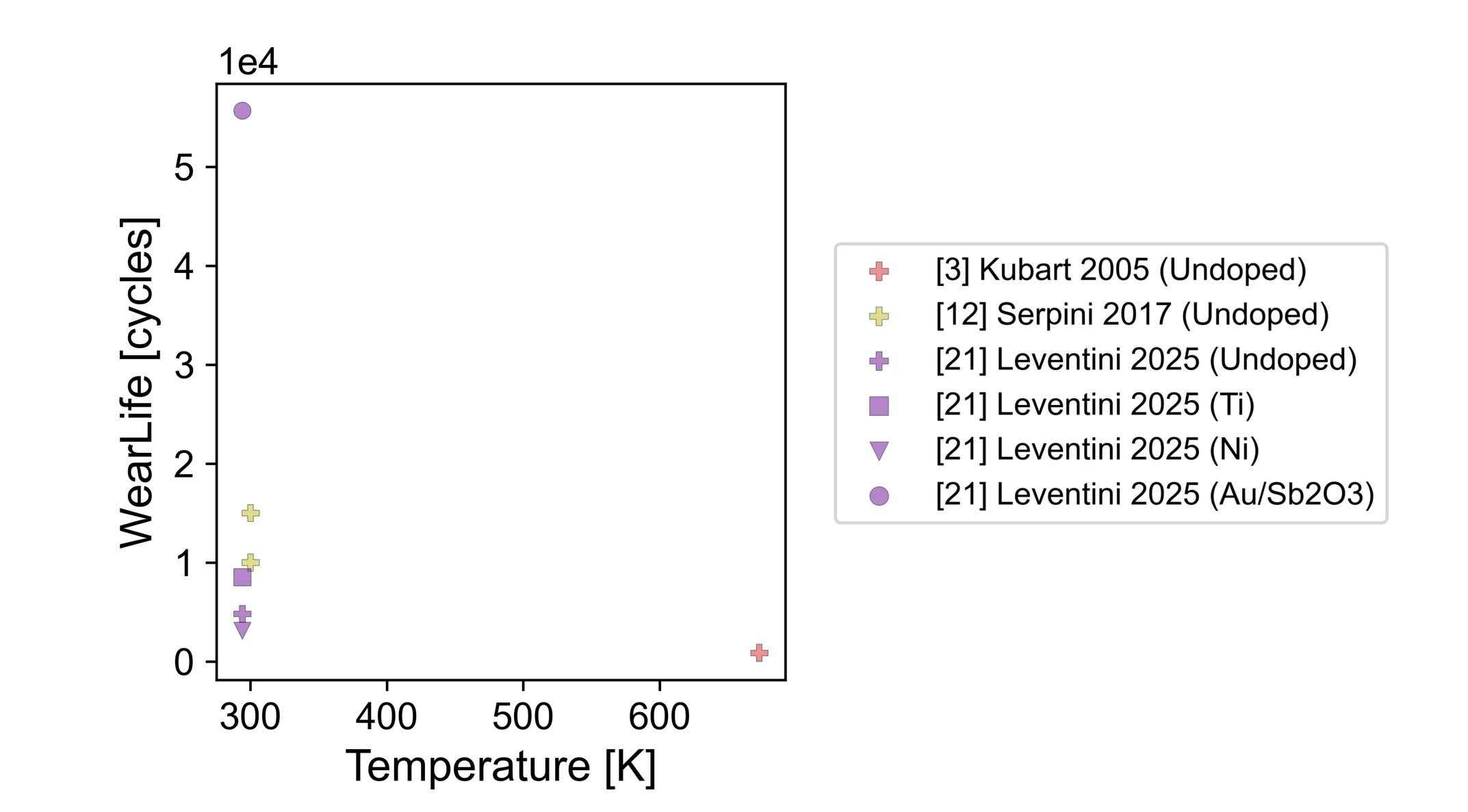


Figure S15: A focused view of results from air conditions below 700 K, showing wear life.


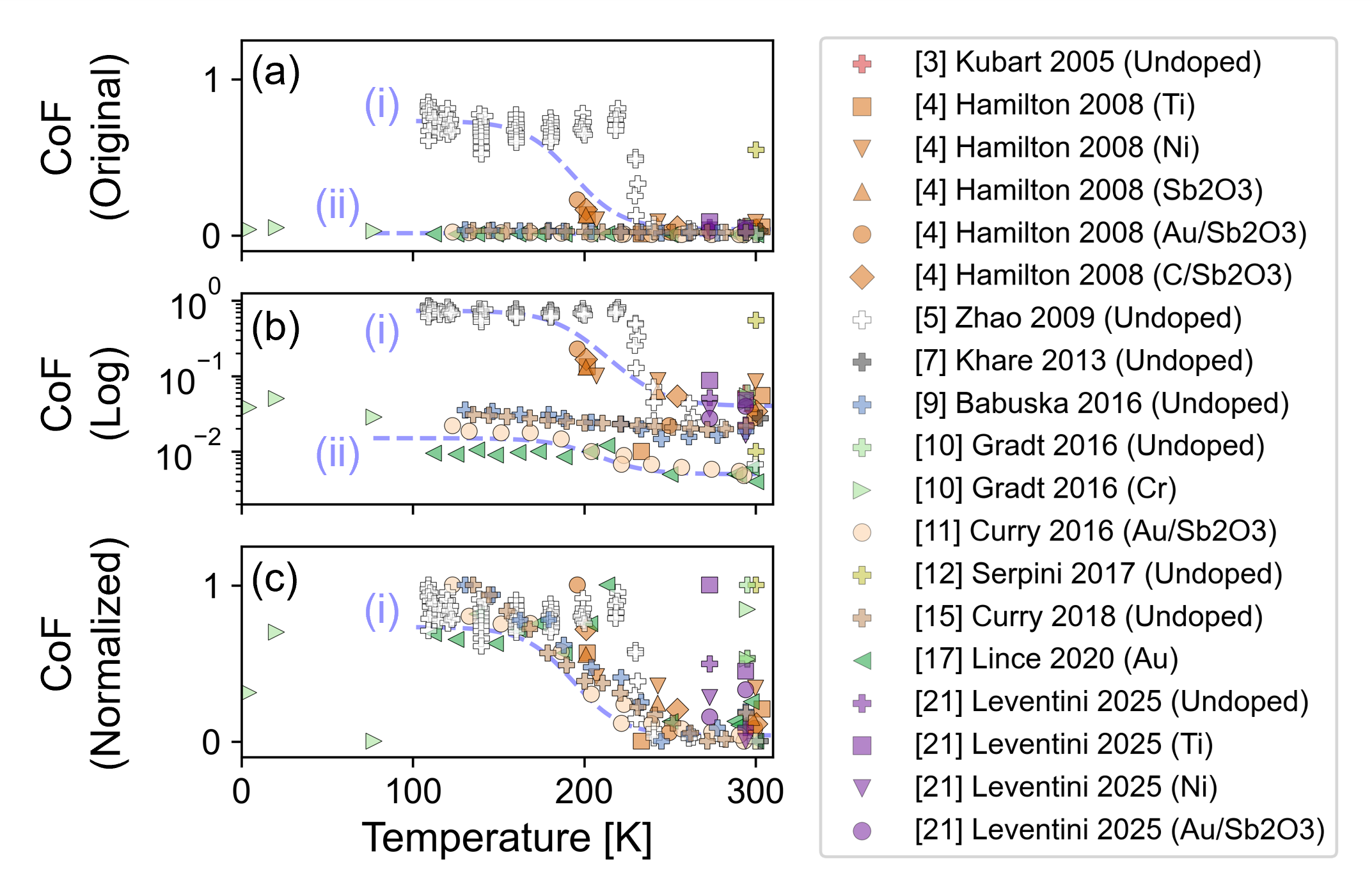


Figure S16: A focused view of results from vacuum or inert gas conditions below 300 K, showing the coefficient of friction in (a) original scale, (b) log scale and (c) normalized scale. In (b), the same trendlines, (i) and (ii), are taken from Figure 1 and are presented for visual aid only rather than from fitting data. In (c), CoF data is normalized for each reference and dopant if more than one dopant is used in a single reference. From (c), all CoF data that crosses the transition temperature, exhibits the transition behavior near 220 K.
